# Diagnostic electron microscopy in human infectious diseases – Methods and applications

**DOI:** 10.1111/jmi.13370

**Published:** 2024-11-19

**Authors:** Michael Laue

**Affiliations:** ^1^ Centre for Biological Threats and Special Pathogens (ZBS 4) Advanced Light and Electron Microscopy, Robert Koch Institute Berlin Germany

**Keywords:** negative staining, patient sample, sample preparation, ultrastructural pathology, ultrathin section, virus

## Abstract

Diagnostic electron microscopy (EM) is indispensable in all cases of infectious diseases which deserve or profit from the detection of the entire pathogen (i.e. the infectious unit). The focus of its application has shifted during the last decades from routine diagnostics to diagnostics of special cases, emergencies and the investigation of disease pathogenesis. While the focus of application has changed, the methods remain more or less the same. However, since the number of cases for diagnostic EM has declined as the number of laboratories that are able to perform such investigations, the preservation of the present knowledge is important. The aim of this article is to provide a review of the methods and strategies which are useful for diagnostic EM related to infectious diseases in our days. It also addresses weaknesses as well as useful variants or extensions of established methods. The main techniques, negative staining and thin section EM, are described in detail with links to suitable protocols and more recent improvements, such as thin section EM of small volume suspensions. Sample collection, transport and conservation/inactivation are discussed. Strategies of sample examination and requirements for a proper recognition of structures are outlined. Finally, some examples for the actual application of diagnostic EM related to infectious diseases are presented. The outlook section will discuss recent trends in microscopy, such as automated object recognition by machine learning, regarding their potential in supporting diagnostic EM.

## 1 INTRODUCTION

Infections still are a major cause of death on this planet with over 13 million deaths per year in the pre‐pandemic year 2019.^1^ Moreover, researchers from different fields expect the numbers to increase because of a higher risk for disease outbreaks or even pandemics due to climate change and increase in population.^2^ Thus, research in human infectious disease diagnosis, pathogenesis, treatment and prevention is relevant. The COVID‐19 pandemic intriguingly supported this conclusion and demonstrated that more research is needed to achieve a better protection of public health against infectious diseases in future.

Diagnostic electron microscopy is an imaging method which allows the visualisation and detection of pathogens, such as viruses, bacteria, parasites and fungi, based on their morphology.^3^, ^4^, ^5^, ^6^ Actually, the development of electron microscopy starting around 1930 was at least partially driven by the wish to visualise viruses which caused relevant diseases at that time.^7^ It was the advent of the early cell biologists in the 50s and 60s of the last century which demonstrated that cellular and subcellular entities possess universal structural signatures which allows their recognition in almost any biological context.^8^, ^9^ This fundamental finding was not only found valid for eucaryotic cells but also for procaryotes, even viruses and led, for instance, to a first systematics of viruses based on morphology.^10^

With the development of new and more robust methods, such as negative staining^11^ and ultrathin sectioning (see Ref. [9] for a summary of the most important developmental steps), electron microscopy became an important diagnostic and research tool in the field of infectious diseases. The method was indispensable for the identification of viruses and other pathogens as the cause for a disease and allowed the clinical differential diagnosis of infectious diseases.^12^ A prominent example for the important diagnostic use of electron microscopy is the fast differential diagnosis between chickenpox and poxvirus infection.^13^ The development of molecular biology and molecular diagnostics, such as selective nucleic acid amplification or sequencing methods, allowed a more precise detection of pathogens, even to the level of individual isolate, than diagnostic EM can provide. As a consequence, the use of diagnostic EM in human infectious diseases declined constantly and today is only applied by few institutions on a regular basis. For animal and plant diseases, the situation is similar although the variety of host species and pathogens is comparably large which limits the broad availability of molecular diagnostic tests or reference sequence information so far.

Nevertheless, diagnostic EM still has its place among the methods diagnosing and studying human infectious diseases. And the simple reason for this is that EM is the only method which is capable of directly visualising small pathogens, that is, the infectious unit, such as viruses, without using any specific probe or staining. This feature is relevant for a couple of critical applications. One is the presence of a serious threat for public health, like an outbreak of an emerging infectious disease or the intentional or unintentional distribution of pathogens in the public space.^3^, ^6^, ^14^, ^15^ Diagnostic EM serves as a fast scouting method for and as an independent control of molecular diagnostics, which both, speed and reliability of diagnostics, are necessary in scenarios of exceptional importance for public health. A second important use case is the study of infectious disease pathogenesis. For understanding the processes induced by the pathogen in the patient, it is necessary to follow the pathogen directly in the spatial context of patient tissue and cells and not only rely on the presence of their molecular traces which can be misleading.

In this review article I am describing the methods which are generally used in diagnostic EM with the relevant variants I am aware of. Moreover, I am drawing attention also to weaknesses and shortcomings which should be addressed in future. Finally, I am describing a few applications of diagnostic EM with the aim to demonstrate the particular relevance of diagnostic EM as outlined above.

## 2 METHODS

The main methods used in diagnostic EM of human infectious disease, negative staining and thin section EM of resin‐embedded samples, are standard in the field of EM for many decades and were only slightly modified or extended over the years. The reason for this comparatively conservative application of methods is mainly that a vast amount of reference data collected in the literature over decades was produced with these rather basic and old methods. A considerable change of the methods would also change the appearance of structures and likely would need new reference material to be produced which is extremely time consuming. Moreover, the basic methods are very robust and reliable although they definitely introduce artefacts which on the other hand could be useful diagnostic criteria if they are reproducible and specific. Today, modern cryo‐preparation and cryo‐EM techniques are the standard for studying many cell biological questions, a strict requirement for the investigation of macromolecular structures, and they provide the best structural preservation.^16^, ^17^ However, there are still many dis‐advantages of cryo‐preparation and especially of cryo‐EM techniques which precludes the application of these methods in many use cases of diagnostic EM. One essential drawback of cryo‐EM in the present state is that it is not very efficient in fast and high throughput screening of samples with unknown and/or low abundant targets, mainly because of the requirements of low dose imaging of frozen‐hydrated samples (see Mills^18^ for a review of the requirements of state‐of‐the‐art cryo‐EM). Another, more basic dis‐advantage of employing cryo‐preparation techniques is that cryofixation requires special equipment which is usually not available at sites where diagnostic samples are collected (e.g. a hospital).

**FIGURE 1:**
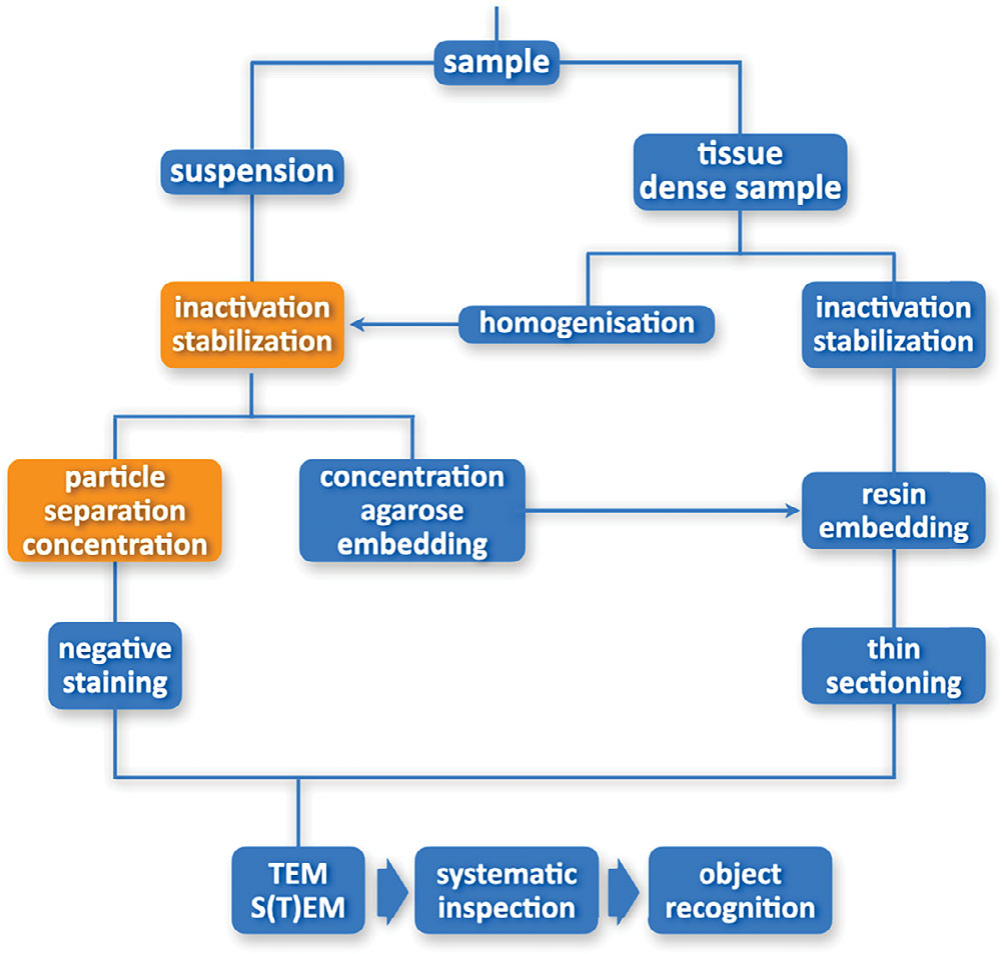
Overview about sample preparation methods usually applied in diagnostic EM. The different preparation steps are arranged as a flow chart starting with the sample and an initial bifurcation into the preparation of the two main sample classes, suspensions and tissue or compact samples. Obligatory preparation steps are shown with blue background, optional steps with orange background.

Figure 1 provides a schematic overview about the basic methods used in diagnostic EM of human infectious diseases focussing on the two relevant sample classes, suspension and tissue or, more general, dense/compact samples. The following subsections describe the methods and process steps mentioned in more detail, starting with negative staining of suspensions and ending with a general consideration on the range of suitable samples for diagnostic EM, their collection, conservation/inactivation and preparation for the application of the EM methods.

### 2.1 Negative staining EM of suspensions

**FIGURE 2:**
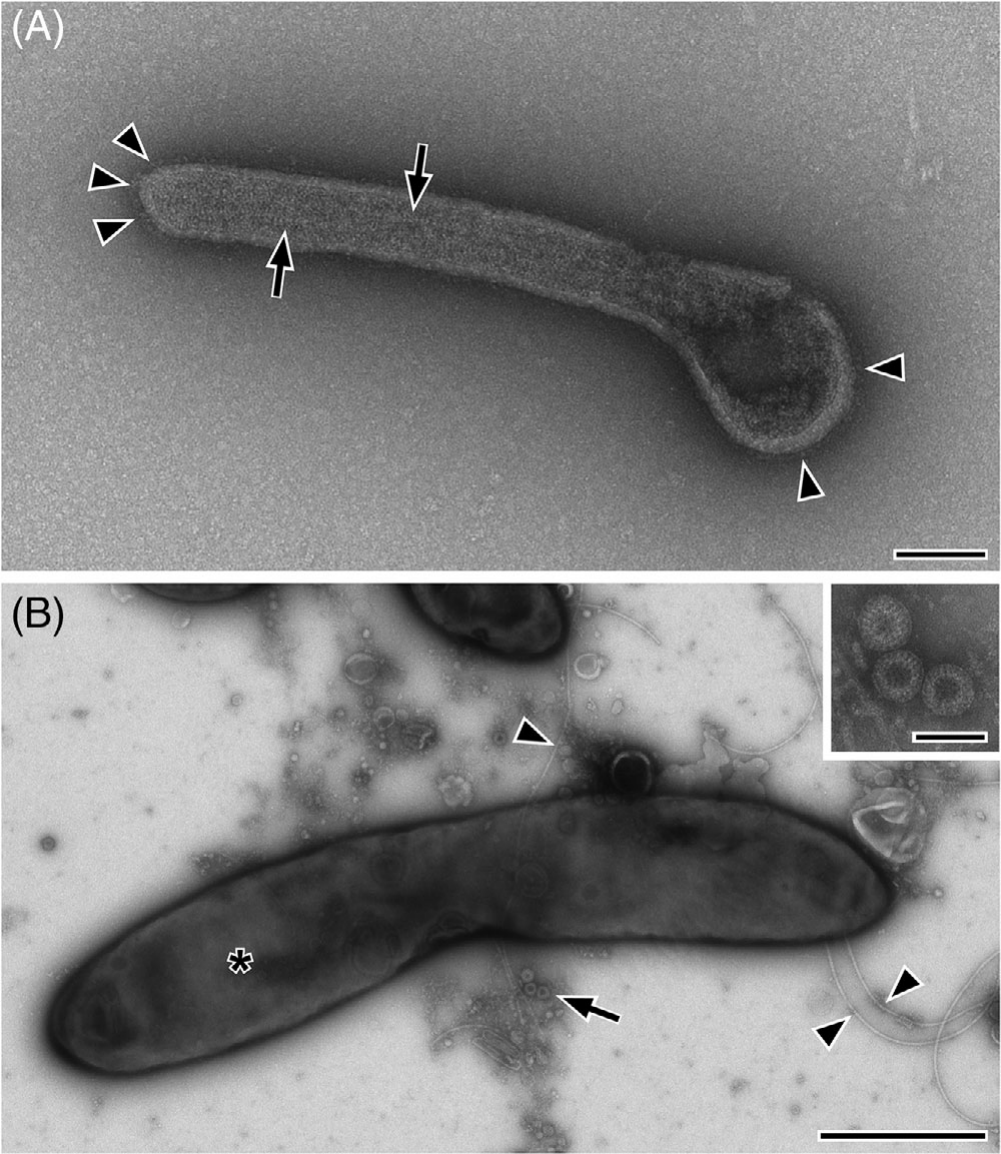
Negative staining electron microscopy. (A) Zaire Ebola virus (ZEBOV) from cell culture stained with 0.5 % uranyl acetate. The filamentous, membrane enveloped virus particle is embedded in a thin layer of dark appearing stain which also has penetrated the virus particle. Surface protein particles are visible at the membrane (*arrowheads*) as well as the nucleocapsid (*arrows*) in the particle interior. The image was recorded by Tobias Hoffmann (Robert Koch Institute). (B) Stool sample from a patient stained with 0.5% uranyl acetate. A large rod‐shaped bacterium (*) is visible in the centre of the image and is associated with some flagella (*arrowheads*). A cluster of Rotavirus particles is localised in close vicinity (*arrow*) and shown in the inset at higher magnification. All images were recorded using a transmission electron microscope (120 kV) equipped with a bottom‐mounted CCD camera and 4k by 4k pixels. Scale bars in A and the inset of B = 100 nm, B = 1 µm.

The negative staining technique is used to visualise small particles of a suspension by transmission EM. Particles are adsorbed on the surface of an electron‐transparent sample support and are embedded in a thin layer of amorphous heavy metal stain. The latter is to stabilises the particle morphology and to highlight biological particles that appear more or less bright (i.e. negative) against the darker heavy metal surrounding (Figure 2). The charged heavy metal ions may selectively bind or associate with certain parts of the particle surface or even penetrate the particle to reveal internal structure of the particle. This generates a stain‐ and object‐specific pattern in the microscope which allows particle recognition if the pattern is unique.

Negative staining was developed in the late 40s to 50s of the last century. The publication of Brenner and Horne^11^ is one of the key publications which clearly demonstrates the benefits and principles of the method using simple viruses as a model. Various modifications for different purposes (e.g. for the analysis of macromolecules) have been developed since that time. Some of them are discussed in monographic books on negative staining which also provide information on historical aspects.^19^, ^20^

In diagnostic EM, a three‐step sample preparation protocol for negative staining is widely used today: (1) particle adsorption, (2) washing, (3) heavy metal contrasting.^21^, ^22^ As sample support, EM grids, usually made of copper, and filmed with an electron‐transparent polymer (Formvar or Pioloform) are generally used. A mesh size of 300 or 400 mesh provides a good compromise between film stability and visibility. Application of a thin carbon layer on top of the polymer film reduces the specimen drift during imaging because thermal energy introduced by the beam is more efficiently distributed across the grid and to the holder than without carbon. It also reduces the risk of introducing holes into the supporting film. An efficient particle adsorption to the polymer or carbon surface of the sample support requires a charged surface which makes the surface hydrophilic and sticky. Physical and chemical methods are used to achieve this. Treatment with a plasma by glow discharge^23^ or UV irradiation is used to pre‐treat the sample support surface without introducing chemical modification of the surface. However, the effect is not stable over time.^21^ The addition of charged chemicals to the surface is an alternative to the application of physical methods. The histological stain Alcian blue, poly‐L‐lysine or the antibiotic Bacitracin were used, among others, to add charges to the film surface.^21^ We prefer using Alcian blue for conditioning of our grids directly before negative staining (see Supporting Information) because it is fast and proved to be extremely robust and reproducible in terms of efficiency in adsorbing particles.^24^ The treatment is at least as efficient as the glow discharge in our hands and seems to capture larger particles, such as bacteria, more efficient than glow discharge.

The particles of a suspension are usually adsorbed at the surface of the grid by adding a small volume of the suspension on top of the pre‐treated surface (Drop‐On‐Grid, DOG) or by placing the grid on a small droplet of the suspension (Grid‐On‐Drop, GOD). For dense particles, such as bacteria or poxviruses, the DOG procedure provides more particles on the grid,^24^ while particles of lower density generally reveal the same particle number on the grid with both adsorption procedures. The GOD procedure can be helpful in samples which contain a high concentration of (irrelevant) particles of higher density because they tend to sediment and therefore are moved away from the sticky surface of the grid during incubation. This reduces the amounts of larger particles bound to the grid film which may mask smaller particles of interest. Therefore, both adsorption procedures are part of our standard procedure for investigating suspensions of unknown content (see Supporting Information).

Incubation time of the suspension at the grid surface has an impact on the number of particles adsorbed. Experiments with an orthopoxvirus and bacterial spores demonstrated that the number of particles increase with time of incubation using the DOG procedure.^24^ While the number of spores seemed to reach a plateau after 20 min, the number of vaccinia virus particles still increased. Similar results have been reported for particle adsorption of other particles, including ferritin and liposomes, adsorbed onto grids used for cryo‐EM.^25^ We decided to use 10 min as standard incubation time, because this time offers a suitable compromise between speed and detection efficiency. Based on the experiments mentioned above, the increase of particle number at the grid surface after 10 min of incubation is only about factor 2 per 10 min during a prolonged incubation which would only slightly affect the detection limit.

Washing of the adsorbed particles removes unwanted solutes, such as phosphates, which interact with the heavy metal staining. However, it also removes particles from the surface.^24^, ^25^ Therefore, the number of washing steps should be limited. We use three brief washes on droplets of de‐ionised water without removing the liquid after each step from the grid surface by using filter paper (see Supporting Information). Before placing the grid on a droplet of the heavy metal stain solution, the water is quickly removed from the grid surface by touching a piece of filter paper to avoid dilution of the staining solution.

A wide range of heavy metal stains is available and used for negative staining EM in low concentrated aqueous solution.^19^, ^20^, ^21^, ^26^ Most widely applied are phosphotungstic acid at neutral pH (actually their sodium or potassium salts; PTA), uranyl acetate (UA) or formate and ammonium molybdate. Today, in infectious diseases, PTA and UA are still widely used and results can be compared directly with published data obtained with the same stains over at least five decades. We apply both stains in our standard protocol (see Supporting Information), because they may provide different information by selectively preserving or showing structural detail which is important for recognition. Moreover, with samples of unknown composition interference of components with the stain is possible. Therefore, a second stain provides a higher chance to obtain information from the sample in the first experimental run. During the last decades methylamine tungstate (MAT) became popular in virus diagnostics and in structural biology for rapid quality checking of protein suspensions. It provides a good compromise between PTA and UA and is available in ready‐to‐use solution (e.g. VitroEase Methylamine Tungstate Negative Stain, ThermoFisher) which, at least in our hands, should be diluted 1:5 with water to avoid crystallisation on the grid.

A critical step of the negative staining procedure is the formation of the thin heavy metal film which finally embeds the adsorbed particles and provides stability and contrast. Many parameters, such as concentration of the stain or particle density at the grid surface, affect the quality of the heavy metal film formation. By blotting the grid at one edge with a filter paper, usually a gradient in film thickness across the grid is produced which allows to find a suitable film thickness for the various particles of a sample during microscopy. Different stains may need different blotting strategies to generate suitable film thickness. Samples stained with PTA usually can be blotted dry while samples stained with UA or MAT should be blotted carefully and leave some liquid at the grid surface. This is usually done by observing the removal of the liquid from the grid during blotting from the side and by lifting the grid from the filter paper when the liquid removal has reached the middle of the grid.

The original negative staining protocol by Brenner and Horne^11^ used mixtures of PTA and virus suspension for direct application on the grid with a final blotting step without any washing steps which was very popular in the last century, at least in virology.^27^ This is the fastest negative staining protocol which minimises the risk of particle desorption during washing. However, it is also prone to unwanted interaction with ingredients of the sample suspension and not compatible with UA.^26^

Negative staining has a couple of limitations. Although the heavy metal staining film stabilises the morphology of labile particles (see images of PLA particles in the description of our standard protocol referenced in the Supporting Information), morphological changes can be introduced by the final drying of the sample. With chemically fixed particles the risk of an entire particle collapse is reduced. However, larger particles, such as bacteria, are usually affected by the drying even in fixed condition. One effect of fixation is that the stain can access the interior of membrane‐enveloped particles much easier which is not always beneficial as it may reveal but also mask details by overloading it with stain. Another important limitation to mention: negative staining needs a relatively high concentration of particles in the suspension to be detected in the microscope. Using rather pure suspensions of an orthopoxvirus and bacterial spores, we could show that the detection limit is about 10^6^ particles per mL using the standard adsorption and incubation protocol outlined above^24^ which is in the range claimed by other authors.^28^

To get more particles adsorbed at the grid surface, various strategies have been developed. Centrifugation and ultracentrifugation are still the most frequently used methods to increase particle density in negative staining EM. If larger volume of a suspension is available, concentration by classical ultracentrifugation is one appropriate method to concentrate particulate material which then is resuspended in small volume and adsorbed at the grid surface.^29^ In addition, gradient centrifugation was used to purify and to enrich suspensions.^30^, ^31^ Smaller volumes can be sedimented using desktop ultracentrifuges, such as the Airfuge (Beckman Coulter).^32^ An efficient variant of Airfuge sedimentation of particles in suspension is to centrifuge the particles directly onto the surface of the grid. For this purpose, a special particle counting rotor^33^, ^34^ or a fixed‐angle rotor,^35^ mostly in conjunction with particular adaptors which the grids are fitted in, are used. Using the latter approach (for a detailed protocol, see Supporting Information), the detection limit could be decreased by at least a factor of 10 (i.e. 10^5^ particles/mL).^24^ Enrichment factors may be higher for suspensions already present at higher concentration.^21^, ^24^ A drawback of all centrifugation techniques is, that all particulate material can be sedimented which may cause an overload of the grid or masking of relevant structures.

**FIGURE 3:**
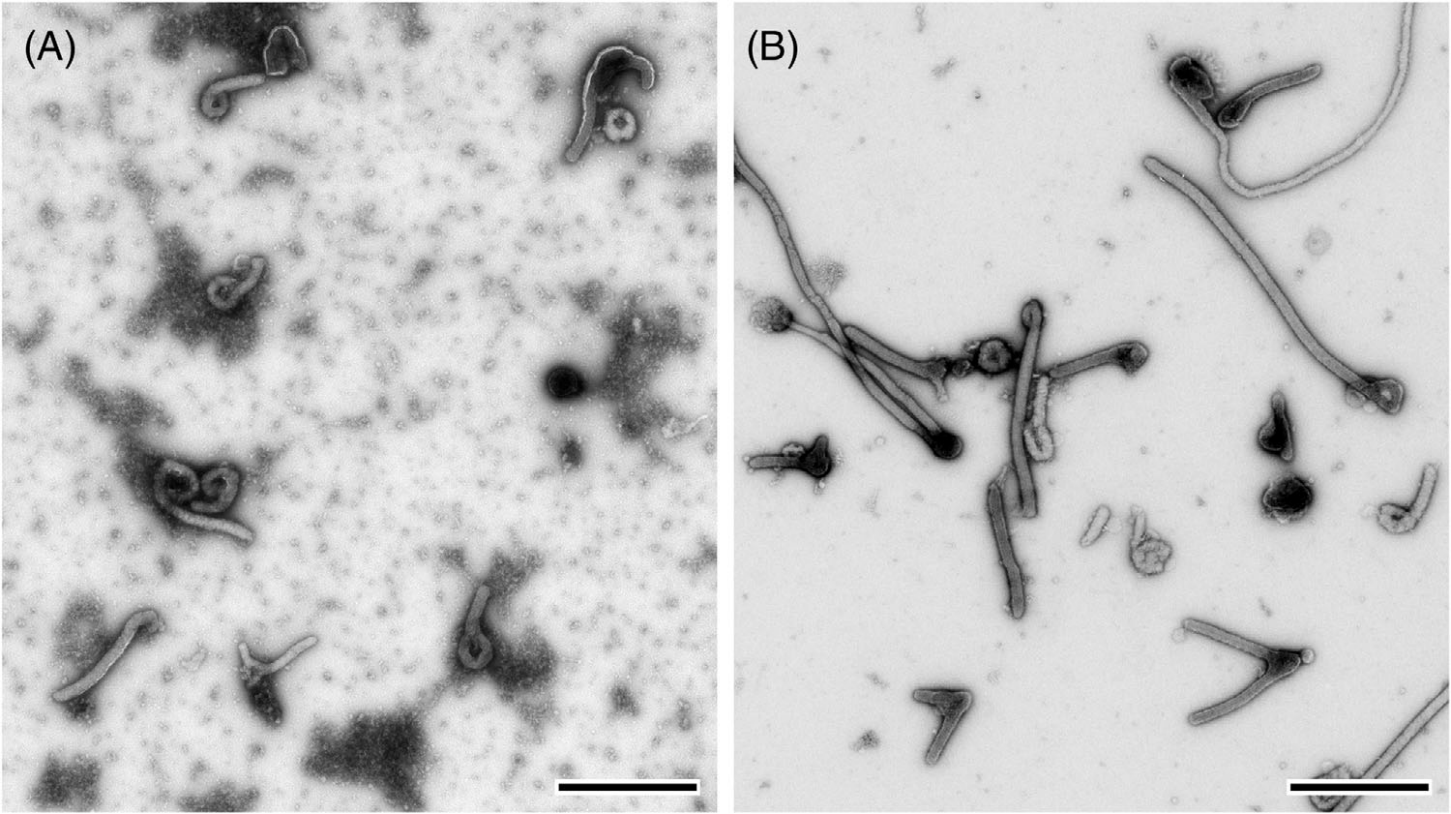
Negative staining electron microscopy of Zaire Ebola virus (ZEBOV) from cell culture stained with 0.5 % uranyl acetate. (A) The cell culture supernatant already shows many typical filamentous viral particles but also smaller particles and their aggregates in the background. (B) After filtration with a nanoporous spin column filter (200 nm pore size), the background was depleted from smaller particles and virus particle density has increased. Sample preparation and imaging was done by Tobias Hoffmann (Robert Koch Institute). The images were recorded using a transmission electron microscope (120 kV) with a bottom‐mounted CCD camera and 4k by 4k pixels. Scale bars = 1 µm.

Filtration techniques were also used to concentrate particles in suspension. The oldest method, which is rarely applied today, is the agar diffusion technique introduced by Kellenberger and Arber.^36^ It was also used for the determination of particle concentration in the tested suspension. The method applies porous colloidium polymer film placed on agar as a filter to retain relevant particles of suspensions and to remove unwanted solutes. A much simpler variant was demonstrated by Anderson and Doane.^37^ Today, nanoporous and molecular filters offer the possibility not only to enrich but also to filter particles selectively from a suspension.^38^ However, selective filtration for negative staining EM has not been evaluated systematically yet, but seems to be promising (Figure 3). An interesting approach to use nanoporous filters was demonstrated by Beniac et al.^39^ The authors used a pumping device to pump suspensions through a filter which carries a grid and demonstrated remarkable enrichment of various viruses and small bacteria. Moreover, the method is independent of the concentration of the suspension. It traps the particles of an entire volume at the surface of the filter membranes with a detection limit of 5 × 10^3^ particles per volume. Most of the filtration techniques work with comparable pure suspensions but were not frequently used for the preparation of clinical samples. One reason might be that clinical samples usually contain a lot of ‘unwanted’ particles which could interfere with the filtration by fast saturation of the filter.

Selective enrichment of relevant particles by using the specific affinity of antibodies for the particles of interest may avoid the overload of the grid with ‘unwanted’ material. Several approaches have been published to use specific antibodies for trapping particles of interest (see Ref. [40] for an overview). One significant problem is that the application of such protocols is limited by the availability of antibodies and it needs already some information about the sample. A more practical method for applications in diagnostic EM is the use of convalescent sera to aggregate viruses.^41^ The idea of this technique is to use the serum of the patients which in many cases quickly produce antibodies against the pathogen to be diagnosed. The serum is then used to aggregate the particles in suspension by the antibodies present in the serum which leads to more efficient sedimentation by gravity onto the grid during incubation. This powerful method is, however, restricted to cases in which suitable convalescent serum or antibody is available.

In our hands, Airfuge enrichment using the fixed‐angle rotor with grid adaptors^24^ (for a detailed protocol, see Supporting Information) provide the best compromise between preparation time, lab safety and enrichment efficiency. Moreover, it seems to be less prone to grid overload than the filtration methods, especially if larger particles or aggregates are present in the sample. This observation needs further systematic validation. Combinatory approaches of filtration, with removal of very large and small particles or macromolecules, followed by Airfuge enrichment may reach a lower detection limit than using the Airfuge alone and, additionally, may avoid overloading a grid or masking of structures.

### 2.2 Thin section EM of dense/compact samples

To analyse the ultrastructure of dense or compact samples without disintegration of their integrity, for example, in cases where information on the pathogenesis of an infectious disease or the cellular tropism of a pathogen is needed, the structure of the sample must be conserved by chemical fixation. Freezing or long‐time storage at refrigerator temperature without fixation should be avoided because it impairs structural preservation (see Section 2.4). Moreover, the stabilisation by chemical cross‐linking of compact biological samples is required because sample preparation protocols use organic solvents which efficiently extract most of the unfixed material (see Ref. [42], chapter 1, for a discussion of this topic and Ref. [43] for the extraction of lipids in chemically fixed cells). For compact biological samples, combinatory fixatives of formaldehyde and glutar(di)aldehyde, such as the popular Karnovsky fixative^44^ (4% formaldehyde and 5% glutar(di)aldehyde) or variants are widely used. The idea of combining the two aldehydes is that the small formaldehyde molecules penetrate much quicker in compact tissue than the larger glutar(di)aldehyde molecules and that the glutar(di)aldehyde cross‐links much more efficiently than the formaldehyde. We use a mixture of 1% formaldehyde and 2.5% glutar(di)aldehyde in HEPES buffer for fixation of tissue samples. The rationale for using 1% formaldehyde is that the equilibrium between monomers and polymers of formaldehyde is comparatively stable (see Ref. [45], chapter 3) at this concentration which allows storing the fixative for a few weeks without fresh depolymerisation of the formaldehyde from para‐formaldehyde directly before use. The buffers used in practice for fixation are variable (see Ref. [45], chapter 3). Cacodylate buffer, which is toxic, still is widely used as it provides suitable preservation and appearance in many samples most probably because it contains charged and uncharged molecules. Phosphate buffers are very popular because they are cheap but do not enter the cells, at least in the beginning of fixation, due to their charged ions. It is important to note that the buffer efficiency of the widely used phosphate‐buffered saline (PBS) is much lower than the capacity of a usual phosphate buffer, for example, according to Sörensen (e.g. Ref. [42], chapter 1), and should be avoided because aldehyde‐fixation with two aldehydes produces considerable amounts of protons which must be buffered to avoid structural impairment. The nontoxic buffers according to Good et al.,^46^ such as HEPES, PIPES or MOPS, are also used for fixation and are rather expensive but popular in cell biology studies. The addition of salts, such as the salts used in the balanced salt solution according to Hanks,^47^ may be helpful to improve structural preservation, especially of membranes (Ref. [48], chapter 2). Systematic studies on the various protocol parameters on structural preservation are missing. Best recommendations are found in comprehensive publications on fixation.^42^, ^45^, ^48^, ^49^

**FIGURE 4:**
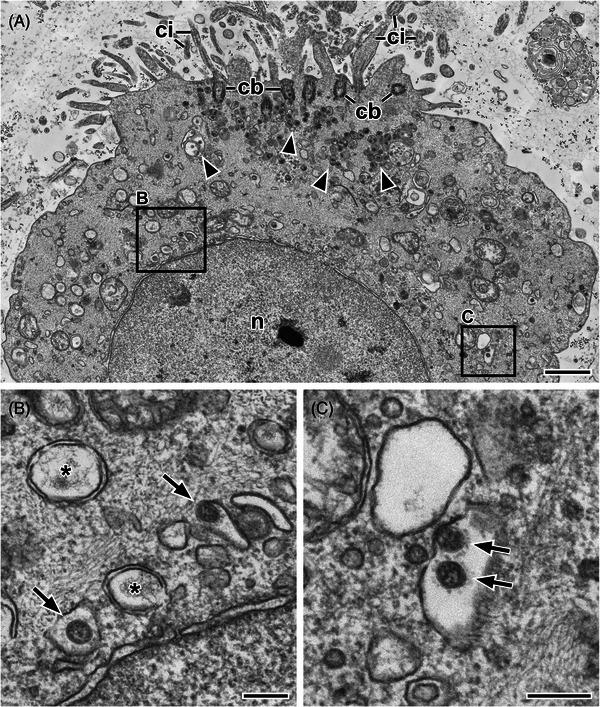
Thin section electron microscopy of naso‐pharyngeal swabs from SARS‐CoV‐2‐positive patients (see Ref. [67] for details of the preparation). Images were recorded with a field‐emission scanning electron microscope at 30 kV using a scanning‐transmission detector. (A) Apical part of an infected ciliated cell present in the swab suspension. Cilia (*ci*) and their basal bodies (*cb*) are clearly visible. Large groups of coronavirus particles, which are always enclosed by an additional membrane, are localised below the basal bodies in the cytoplasm (*arrowheads*). (B, C) Higher magnification of the cytoplasmic regions indicated in A by the respective rectangles show coronavirus particles (*arrows*) and the typical replication organelles, double‐walled membrane vesicles (*). *n* = nucleus. Scale bars in A = 1 µm, in B and C = 200 nm.

All sample preparation protocols for chemically fixed tissue/compact samples usually comprise the following steps: (1) post‐fixation, (2) dehydration, (3) infiltration with resin, (4) embedding and polymerisation, (5) ultrathin sectioning, (6) on‐section‐contrasting. The standard post‐fixation step of many if not all protocols used in diagnostic EM of infectious diseases is incubation with osmium tetroxide (usually at 1–2%). The fixation with osmium tetroxide stabilises membranes because it reacts with unsaturated lipids.^50^ Moreover, it selectively adds heavy metal atoms to the sample providing a basic contrast for imaging. The application of osmium tetroxide needs no particular additives (e.g. buffer), because the primary fixative usually renders the sample osmotically inactive and already provides a certain protection against other chemical impairment, such as proteolytic cleavage which is described for osmium tetroxide treatment.^50^ Optionally, further post‐fixation steps are included in the protocol. Block‐contrasting with uranyl acetate increases the contrast of the sample.^51^ We additionally use tannic acid between osmium tetroxide and uranyl acetate incubation^22^ to further improve the contrast of membranes^52^ and of glycoproteins.^53^, ^54^ This treatment helps to discriminate the various membrane‐bound compartments from each other and to visualise their changes caused by infection (Figure 4). Moreover, it is particular helpful to enhance critical structural elements, such as the surface proteins of membrane‐bound viruses (Figure 4) or cell wall structures of bacteria.

Dehydration is usually performed by using ethanol or, more rarely, acetone in an ascending concentration series. Long‐time storage or incubation should be avoided because both organic solvents extract material from the sample.^43^ For embedding, usually epoxy resin is used because it likely provides the best structural resolution in thin resin section EM.^55^ Many epoxy resin variants are available and were used in the field, for example, the original recipe of Luft,^56^ using Epon 812 (today, substitutes, such as Embed 812, are available), Araldite, Spurr mixture (ERL 4206) or Durcupan (for overview, see Ref. [42], chapter 2). All of these resins are used as more or less viscous monomer mixtures with hardener/softener and accelerator. They are introduced into the sample by using mixtures of the monomer with organic solvents, mostly acetone. In former days, the more efficient solvent propylene oxide was used for infiltration. However, it is toxic and carcinogenic and most likely an efficient extractor of yet unfixed tissue components and, therefore, should be avoided. However, for tissue or objects which are difficult to infiltrate, it may still be useful. The final embedding of the infiltrated material is usually done in capsules or moulds which allow a simple release of blocks after heat polymerisation in an oven. Incubation times for the different incubation steps are largely based on trial‐and‐error and are often longer than necessary which most likely is an advantage because sample size and compactness usually is not very well standardised and therefore even larger samples can be successfully prepared without changing the protocol.

Using an automated tissue processor may help to reduce the workload for sample preparation to a certain extent. However, tissue processors are expensive and also need some time to set up and to clean after work. Moreover, the protocol needs adaptation of the number of incubation steps, because the sample carriers usually transport considerable amount of incubation solution to the next vial. I have added our protocol for using the Leica TP to the Supporting Information. An alternative for using an automated tissue processor may be the use of segmented sample containers with manual transfer which allow to incubate several samples in one vial. This reduces the amount of pipetting and/or manual transfer steps.

Paraffin‐embedded material can be used for thin section EM and provide a helpful guidance for ultrastructural analysis (see Section 2.3). However, the samples are usually of much lower structural quality than material which was directly prepared from fixed material.^57^, ^58^, ^59^ The main reasons for this are that the paraffin samples are usually fixed with formalin which is less efficient than fixation with formaldehyde or formaldehyde/glutar(di)aldehyde mixtures and that the paraffin embedding leads to extraction and structural impairment. This impairment usually has consequences for the recognition of structural features and generally makes it more difficult (see Section 2.3). Paraffin sections or blocks can be used for EM sample preparation (see also the mpox case described in Section 3). Sections are processed on slides and material from paraffin blocks can be extracted by using a tissue punch. Before de‐paraffinisation with xylene, most of the paraffin must be removed. After the de‐paraffinisation and rehydration, samples should be fixed with glutar(di)aldehyde (e.g. 2.5% in 0.05 M Hepes buffer). Finally, the standard embedding protocol for thin section EM can be performed (see Supporting Information for a protocol). Sections on slides are embedded in situ by using inverted embedding capsules^60^ or covered by a thin layer of resin^61^ and released from the slide after polymerisation.

The usual processing time of about 2–3 days for embedding and polymerisation might be too long in certain cases (e.g. outbreaks of highly pathogenic and transmissible pathogens). Thus, rapid protocols for embedding have been developed. The easiest approach to shorten the processing time is to increase the temperature for polymerisation of the epoxy resin which reduces the time for hardening the sample blocks to a few hours.^62^, ^63^ If a further reduction of the sample processing time is necessary, for instance to allow a same‐day diagnosis, the entire protocol must be shortened which is probably best done by using microwave assistance. While the number of processing steps in most protocols remain the same as in a standard embedding protocol, the incubation times are significantly reduced because of the higher temperature applied by the microwave which increases reaction speed and diffusion. This allows processing times of a few hours.^64^, ^65^ To reduce the time for embedding further, around 1 h including polymerisation, requires an entirely different approach. We achieved this goal by using thin samples in conjunction with rapid embedding in LR White which can be polymerised at high speed using a chemical accelerator.^66^ However, the structural resolution and preservation of the samples are different after rapid LR White embedding than after standard epoxy resin embedding but sufficient to detect relevant detail for recognition of ultrastructures and pathogens.^22^, ^66^, ^67^

Thin section EM of suspensions might be necessary in some cases, for example, if the negative staining of a suspension has revealed dense, electron intransparent objects of putative biological origin that need to be identified. Suspensions with high concentration of target particles can be directly mixed with low‐melting point agarose and casted in a thin gel^66^ (see Supporting Information for an actual protocol). To achieve higher concentration, ultracentrifugation can be used and the pellet can be resuspended in a small volume of low‐melting point agarose that is solidified on ice for further processing. If only small sample volume is available, Airfuge sedimentation can be used with an inert stain (i.e. colloidal gold particles) to localise the tiny pellets.^67^ They are recovered from the vials by covering them with low melting point agarose and enclosure in more agarose before subjecting them to standard processing for thin section EM. This procedure allowed to visualise shed cells, secretions, bacteria and coronaviruses in nasopharyngeal swabs of patients infected by SARS‐CoV‐2 (Figure 3).^67^

Ultramicrotomy of embedded samples follows standard procedures. For large‐field recording of ultrathin sections (see Section 2.3), special requirements must be considered.^68^, ^69^, ^70^ On‐section contrasting with uranyl acetate and lead citrate is usually applied to increase the visibility of relevant structures. It can be avoided in samples which were already treated with several heavy metals or a high concentration during sample preparation. However, it should be evaluated if the contrast is sufficient for reliable object recognition with the available electron microscopes and detectors.

### 2.3 Examination of samples by EM and object recognition

A reliable assessment of a sample requires that all relevant structural elements are conserved during sample preparation and that the inspection of samples at the microscope allows their identification. This trivial statement implies that a couple of aspects are considered. First of all, the sample must be chemically fixed or kept in a form that limits structural change to a minimum until examination. Second, the selection of sample regions for inspection should be as representative as possible. Third, the microscope must be properly aligned, calibrated for correct magnification (correct size measurements are needed for a correct identification of certain objects, e.g. small viruses) and should allow the visualisation of the structures of interest at a suitable resolution. Last but not least, the operator must be able to recognise all relevant structural details of the sample to finally assess the sample.

The conservation of structural elements of a sample during sample preparation until the final inspection at the microscope is not a simple task. Fixation of samples early in the process stabilises a sample and preserves structural detail until inspection (see Section 2.4). Sample preparation protocols should be standardised and checked for their reliability by using reference material with known structures (e.g. cultures of the target pathogen). Sampling of available samples for imaging is critical for the outcome of an investigation. Homogenised suspensions are comparatively easy to investigate, because every portion of the suspension should contain the same particles at the same concentration. With a fixed number and mode of screening of the grids prepared, a constant detection likelihood should be achieved which guarantees that the detection limit of the method can be attained.^24^ In our routine approach, we inspect at least 20 meshes of a 400‐mesh grid (which has about 1200 meshes!) at mid magnification and select regions in these meshes for a detailed screen at high magnification. By applying this procedure, we usually detect small microorganisms present at concentrations down to the determined detection limit.^24^ To control the adsorption and possible interference with the staining, we add a concentrated Vaccinia virus suspension of known particle number to the sample and assess if the control virus can be detected at the expected particle density. This procedure provides an experimental proof whether we can expect to reach the detection limit with the sample or not. Generally, we prepare four grids per sample (two different stains and adsorption variants) and decide if further particle enrichment is required and possible, or not, if the inspection was negative. If suspicious dense objects are detected, thin section EM of the suspension is considered (see Section 2.2).

To sample tissue blocks embedded in resin by sectioning is a much more difficult task than sampling a suspension for negative staining EM. The reason is that pathogens are not necessarily distributed homogenously in the sample block and that ultrathin sections are a comparatively small sample which represent only a tiny fraction of the entire sample block. Thus, any information on the distribution of the pathogens or regions of interest in the sample block will support the investigation. Inspection of paraffin sections may provide such information and paraffin embedded material can be used for further analysis by EM. However, structural preservation usually is impaired in comparison to samples directly prepared for diagnostic EM.^57^, ^59^ If no information on the distribution is available, the inspection of sections from different regions and sample blocks should be considered. I am not aware of any systematic study of the sampling problem. In an unpublished study, we investigated systematically a wide range of tissue from a systemic poxvirus infected elephant. From PCR we knew that the various tissue samples were highly positive for poxvirus DNA. We sampled three different regions (with 200 µm distance) of each resin block which contained a piece of tissue of about 1–2 mm in length. By using this strategy, we were able to find poxvirus particles in every resin block in at least one region. However, this result indicates that finding small pathogens in patient tissue by ultrathin sectioning is difficult and requires some efforts in sample screening. This conclusion is supported by examples from the literature where especially infections with viruses can be focally localised although symptoms, such as inflammation or tissue damage, indicate a more widespread distribution.^71^, ^72^

Imaging in diagnostic EM was usually performed with transmission electron microscopes because speed and spatial resolution were much higher than in scanning‐electron microscopy. During the last decade, this has changed, because modern field‐emission scanning electron microscopes provide a high spatial resolution and are equipped with sensitive detectors to allow acceptable imaging speed for most purposes of diagnostic EM.^69^, ^70^, ^73^ However, transmission‐electron microscopes still allow faster imaging at better resolution than scanning electron microscopes which is relevant for the detection of small viruses (i.e. below 100 nm). Automated imaging of larger areas is particularly interesting for recording entire sections or section areas of ultrathin sections from tissue samples.^68^, ^69^, ^70^, ^72^, ^74^ Such data allow post‐recording analysis by several users at any time. Moreover, it conserves the structural context of a finding at much higher quality than a conventional documentation by overview and detail images. Mostly, scanning electron microscopes are used for the recording of large‐field data sets, but special software solutions, such as Serial EM^75^ or the commercial MAPS (ThermoFisher), allow large‐field imaging with transmission electron microscopes. The website nanotomy.org, founded by Ben Giepmans,^74^ provides an open repository for large‐field EM data, including some data sets from infected tissue or cells. As an example, Video S1 demonstrates the zoom and pan visualisation of a large‐field recording of an ultrathin section from a *Treponema*‐infected skin tissue.

Object recognition is the final goal of any microscopy investigation to allow answering scientific or diagnostic questions. A central requirement for object recognition is the availability of a validated structural reference. In diagnostic EM, mainly books and few digital formats with images of reference structures provide a basis for object recognition (a list of some reference publications is given in the Supporting Information). However, individual and continuous training using suitable samples is required. During the COVID‐19 pandemic, it became evident that the ability to recognise coronavirus particles properly is hardly present. A review of published ultrastructural investigations revealed that in only 9 of 144 publications sufficient structural detail and criteria were demonstrated for a valid identification of coronavirus particles.^76^ Moreover, in many publications even the structural identification of the usual cell organelles and subcellular objects was insufficient and led to misinterpretations.^76^, ^77^, ^78^, ^79^ This was at least partially due to a lack of knowledge but also due to the impaired structural preservation of most of the samples which were acquired from autopsies. However, if specific structural detail is not visible (i.e. all necessary structural elements that are in summary unique for the object), for whatever reason, a diagnosis on the presence of a pathogen using structural features is impossible. In summary, recognition of pathogens and the structural background, which is normally present in the target cells and tissues, needs to be learned and continuously experienced by practicing.

A suitable support for an uncertain identification by structural criteria may be provided by antibody labelling if the specific antibodies are available. Protocols for both, negative staining and on‐section EM, are available^21^, ^22^, ^66^ and allow a comparatively fast response. Recent applications in identifying SARS‐CoV‐2 in samples of the lung^80^ or hepatitis E virus in the seminal fluid^81^ demonstrated the suitability of the combined approach.

Object recognition in our days is more and more performed by procedures using artificial intelligence, mainly machine learning. While this approach is already used in some diagnostic disciplines,^82^, ^83^ automated pathogen detection in diagnostic EM is only in its infancy. So far, only proof‐of‐concept studies were published for the automated detection of virus particles from suspensions^84^, ^85^, ^86^ and for the detection of the different intracellular stages of herpesviruses.^87^ They demonstrate that it is generally possible to detect virus particles in rather ideal samples with a high reliability. However, a validation using patient samples is still lacking and probably would need more efforts in the training of machine learning algorithms. A blueprint for the entire automation of the imaging and classification process was presented for a task of quality assessment using rather purified virus suspensions.^88^ In principle, modern electron microscopes are already able to record images automatically from a sample. The bottle neck for automated object recognition is the availability of suitable annotated reference material for the training process. To solve this problem, more annotated data sets should be published in suitable repositories, such as the BioImage Archive (https://www.ebi.ac.uk/bioimage‐archive/) or Zenodo (https://zenodo.org/), to provide persistent reference.

### 2.4 Sample collection, preparation, inactivation and stabilisation

Sample selection and preparation for an analysis by the methods described above may already have an influence on the outcome of a diagnosis or analysis and, therefore, should be carefully considered on the basis on all available information (e.g. patient anamnesis, role of EM in the actual case). Unfortunately, no systematic studies on this important aspect are published to my knowledge. Most recommendations are based on the experience of persons working in the field for several years.^28^, ^89^, ^90^, ^91^ I have compiled some basic recommendations for collection and preparation of the most relevant clinical sample classes in the Supporting Information. Again, all of these recommendations lack a substantial validation and are based on experience or best guess.

In most cases, transport of the samples from the site of collection to the EM lab is necessary. This should be done as fast as possible and without heating or freezing. Suspensions can be transported without prior fixation. However, fixation with concentrated buffered formaldehyde^92^ stabilises the particles of the suspension for the transport avoiding possible microbial growth and changes of particle structure. Tissue samples which are intended to be prepared for thin section EM should be conserved by chemical fixation as fast as possible, best at the site of sample collection. Buffered formalin can be used and is available in most hospitals. We prefer a mixture of 1% formaldehyde and 2.5% glutar(di)aldehyde in HEPES‐buffer (see Section 2.2) because this is a stable mixture which can be shipped at room temperature and stored for a couple of months (see Section 2.2). Formalin‐fixed tissue should be additionally fixed with glutar(di)aldehyde before starting with the sample preparation for thin section EM.

Autopsy tissue is a special class of samples. While biopsy samples from patients usually are structurally well‐preserved if chemically fixed directly after extraction,^93^, ^94^ autopsy tissue usually reveals structural deficiency which is related to postmortem changes before stabilisation by chemical fixation.^76^, ^79^ Using biopsy systems to quickly extract samples from recently passed away patients may be a strategy to shorten the time between cell death and fixation and therefore improve the structural preservation of the extracted tissue.^71^ However, further research and efforts are needed to generally improve sample collection from autopsies for diagnostic EM of infectious diseases.

Biosafety is an important aspect when working with potentially infectious samples. Legal rules and their application vary from country to country. Therefore, it is not possible to provide distinct recommendations. In most countries, patient samples, which are potentially infectious, should be processed under BSL‐2 conditions. All noninactivated material is processed in a BSL‐2 cabinet, if it is not tightly enclosed. We usually inactivate the material by using aldehydes, if the samples are associated with severe disease or death. Alternative inactivation procedures, such as UV radiation, X‐ray or cold plasma treatment, are not generally validated either regarding safety or their ability to preserve structural features necessary for diagnostic EM. For suspensions, we validated the fast inactivation with 2% formaldehyde by using three model viruses.^92^ The formaldehyde fixation usually does not affect the detection of viruses by negative staining as has been discussed before.^95^ However, fixation may allow stain penetration in larger membrane‐bound particles, such as bacteria, and interfere with the recognition of details. Inactivation of pathogens in cells and tissue, unfortunately, is not well validated so far, perhaps because the samples can be much more variable in terms of size and density than suspensions. Only a few papers systematically address this issue so far, mostly using selected viruses or microorganism.^96^, ^97^, ^98^, ^99^, ^100^ From these studies, we can assume that the usual 1–2.5% of glutar(di)aldehyde is sufficient to inactivate many viruses, if applied on small tissue pieces and for several hours. We usually process all fixed tissues in a BSL‐2 cabinet until reaching the begin of post‐fixation (see Section 2.2) and transfer the samples into fresh vials with fresh fixative. This helps to avoid spread of non‐inactivated fluids from the original vials and of aerosols during liquid handling or tissue manipulation (e.g. cutting into smaller pieces). If risk group 3 or risk group 4 pathogens are (likely) involved, samples will be treated in respective laboratories. Initial fixation will be done with the fixation procedures mentioned in Section 2.2. All of the BSL‐3 and ‐4 labs usually have approved protocols and standard procedures for inactivation of tissue samples (e.g. for standard pathology or light microscopy) which they can use after having applied the fixation for EM to comply with their rules and to allow export from the lab. The first fixation step usually defines the structural preservation of the sample and additional fixation usually does not change it.

## 3 APPLICATIONS OF DIAGNOSTIC EM

**FIGURE 5:**
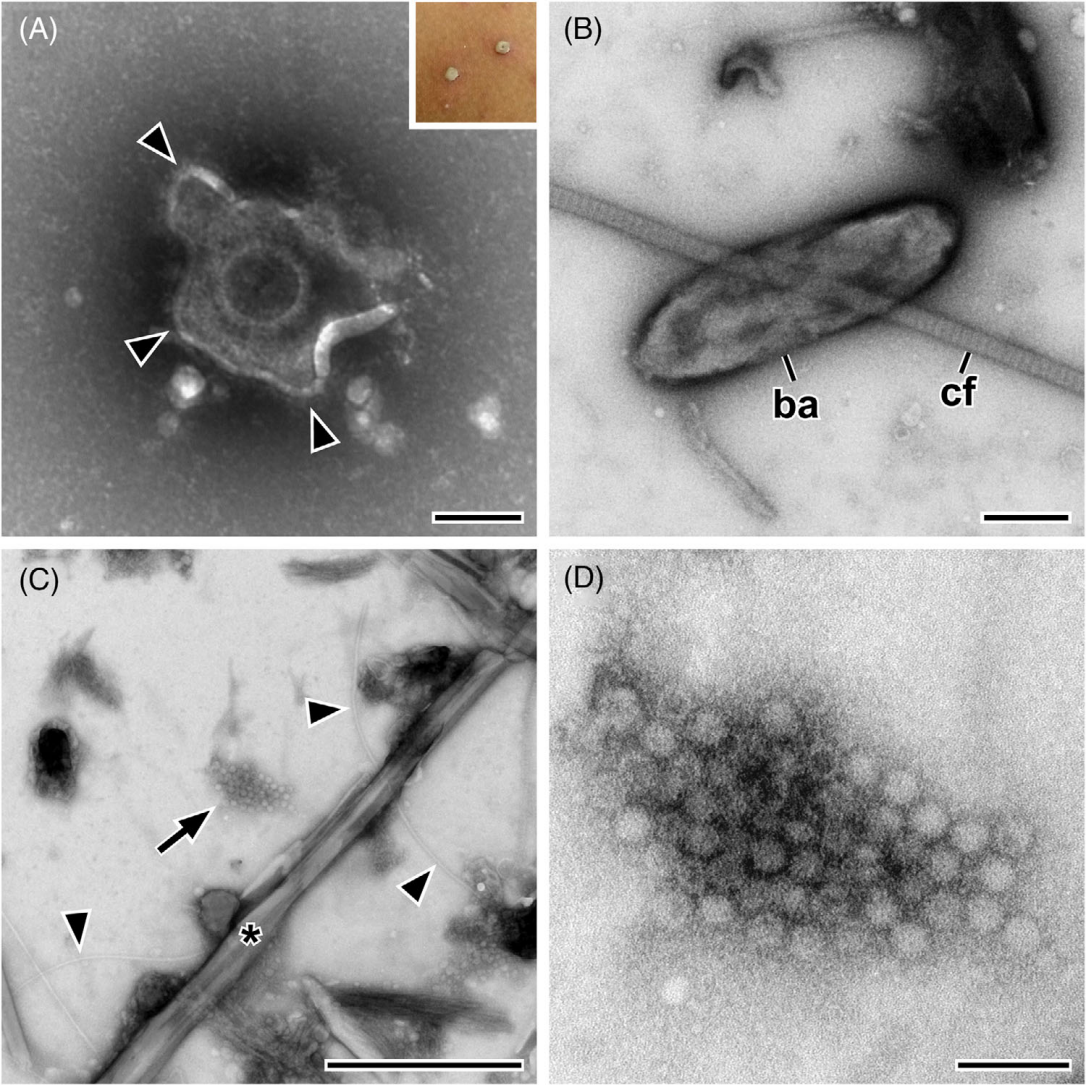
Application of diagnostic electron microscopy in emergencies. (A) Negative staining electron microscopy (1% phosphotungstic acid) of skin‐blister fluid revealed the presence of many herpesviruses (*arrowheads* indicate the enveloping membrane) which supported the possible symptomatic differential diagnosis of chickenpox. The *inset* shows two of the many skin blisters that were visible on the thorax of the patient. The image was recorded using a transmission electron microscope (200 kV) with a side‐mounted CCD camera and 2k by 2k pixels. Sample preparation and imaging was done by Lars Möller (Robert Koch Institute). (B) Negative staining electron microscopy (1% uranyl acetate) of a swab from a treated skin lesion, suspected to contain poxvirus, which was negative except of bacteria (*ba*) which may indicate a bacterial wound infection that should be reported. *cf* = collagen fibril. (C, D) Negative staining (0.5 % phosphotungstic acid) of a stool sample from the large food‐borne gastroenteritis outbreak in Germany, 2012. (C) Overview of a sample region with a cluster of norovirus‐like particles (*Caliciviridae*; *arrow*). Besides the virus particles, crystal‐like structures (*) and bacterial flagella (*arrowheads*) can be recognised. (D) Higher magnification of the virus particle cluster. The images shown in B to D were recorded using a transmission electron microscope (120 kV) with a side‐mounted CCD camera and 1.3k by 1k pixels. Scale bars in A and D = 100 nm, in B and C = 1 µm.

Today, diagnostic EM of infectious diseases is mainly used to support two important tasks in public health protection: (1) diagnosis or exclusion of rare, uncommon or emerging infectious diseases and (2) the investigation of disease pathogenesis. In the past, diagnostic EM was used, like every other medical lab method, to support symptomatic diagnosis by medical doctors.^5^, ^12^ Today, molecular assays, such as the detection of specific pathogen nucleic acids by polymerase‐chain reaction (PCR) amplification, are available which can provide precise information on the identity of a pathogen at highest detection sensitivity. However, the support of symptomatic diagnosis of infectious diseases by diagnostic EM still is of importance in cases where a fast decision is needed to protect public health.^6^, ^14^, ^15^ One example to illustrate this is the case of a couple of refugees from north Africa which were living in a residential home with many people and developed skin blisters over a wide area of their body (Figure 5A). The fast diagnosis by the medical public health officer was not decided between chickenpox or an early poxvirus infection and therefore a small amount of blister fluid was collected and analysed by negative staining EM. Blister fluid from an acute infection usually contains a high concentration of pathogens and therefore a diagnosis at the microscope usually is extremely fast and simple. In this case, herpesviruses were detected within a few seconds of screening the sample (Figure 5A) which supported the possible symptomatic diagnosis of chickenpox and allowed to apply suitable countermeasures within a few hours after first patient contact. Molecular testing is also very fast today and could have answered the question with only slight delay in comparison to diagnostic EM. However, generic methods, such as nucleic acid sequencing, still need at least a couple of hours to provide a result. And this is exactly why fast diagnostic EM still is in use. It can be fast, providing a result within an hour, and is able to see the unexpected if present at sufficient concentration. If none of the two anticipated symptomatic diagnosis, chickenpox or poxvirus infection, would have been correct, diagnostic EM could have provided the negative result or a first hint towards an alternative diagnosis (Figure 5B).

The generic ‘open view’ capability of diagnostic EM supports the diagnosis of infectious diseases in various cases.^5^, ^12^, ^15^, ^101^ One important application is the diagnosis of the pathogen responsible for a disease outbreak relevant for public health. In such cases, usually several methods are used in parallel to find the cause for the observed disease outbreak. In a large gastroenteritis outbreak in Germany in 2012, over 10,000 school children were affected within 2 days. Quickly, the presence of the usual pathogens responsible for causing the observed forms of gastroenteritis were checked by PCR‐based testing. In many samples a norovirus showed up as the responsible pathogen. Since the responsible authorities wanted to make sure that this was the single cause for the outbreak, a test panel with PCR‐positive and PCR‐negative stool samples were provided for diagnostic EM. The diagnosis of the norovirus could be supported for all PCR‐positive samples and no uncommon microorganisms could be detected in the samples (Figure 5C and D). In this case, diagnostic EM provided control for the other diagnostic tests applied and allowed to render the participation of other relevant microorganisms involved in the outbreak unlikely. The epidemiology of the outbreak finally could make out one source for the infection, a contaminated fruit preparation which was shipped by a single distributor to many different school kitchens in the eastern federal states of Germany.^102^

An important group of cases for the application of diagnostic EM are infections in immune‐compromised patients,^3^ because these patients can acquire infections with a wide spectrum of pathogens^4^ and may develop uncommon symptoms. The open view of diagnostic EM can help to find the cause for the infection and may show the way for disease treatment. While this application was widely used in the beginning of the AIDS pandemic, it has shifted towards patients that received donor organs from other individuals, cancer patients or patients with a suppressed or impaired immune system.^103^, ^104^, ^105^, ^106^

The most obvious application for diagnostic thin section EM is the extension of the spatial optical resolution of the histopathology. If the histopathologist identifies tissue and cellular modification indicating the presence of infectious microorganisms which are too small to be resolved by light microscopy, examination by thin section EM can help identifying the pathogen. Common cases are found in dermatology in which the histopathology of skin lesions may show more or less typical structural signatures of a poxvirus infection, that is, the ballooning necrosis and/or typical cytoplasmic inclusions in enlarged epidermal cells.^107^, ^108^ However, the resolution of the light microscope is too low to reveal the poxviruses. Using the de‐paraffinisation and re‐embedding methods described in Section 2.2, the same sample or a sample region close to the region inspected by light microscopy can be investigated by thin section EM and clearly prove the presence of poxviruses within the cellular context.^108^ Moreover, a morphological differentiation between para‐ and orthopoxviruses is usually possible.^13^

**FIGURE 6:**
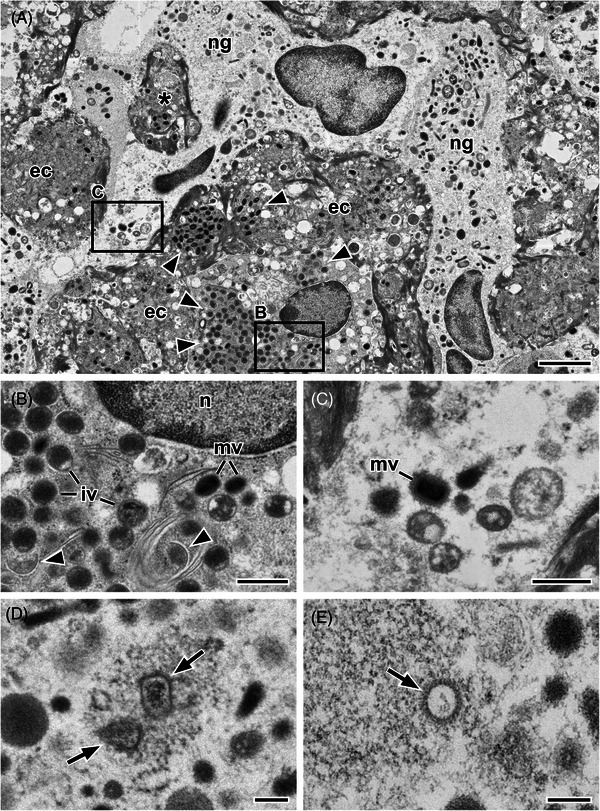
Thin section electron microscopy of a biopsy from the colon of a patient which was infected with the Monkeypox virus (*Orthopoxvirus monkeypox*). (A) Overview about infected enterocytes in a colon crypt. Infected cells contain large clusters of mature and immature virus particles (*arrowheads*). Neutrophilic granulocytes (*ng*) surround the enterocytes (*ec*) and engulf residual infected cell fragments (*) but are devoid of poxvirus replication structures and immature virus particles. (B, C) Detail of A, as indicated by the respective frames drawn in A. (B) Mature (*mv*) and immature (*iv*) orthopoxvirus particles close to the nucleus (*n*) of an enterocyte indicate virus replication. The presence of typical early replication structures of orthopoxvirus replication, the so‐called crescents (*arrowheads*) support this conclusion. (C) A single mature virus particle (*mv*) in the cytoplasm of a neutrophilic granulocyte. (D, E) Pox virus particles in two different neutrophilic granulocytes which are in the decoating stage of infection (compare Ref. [109]). Images shown in A–D were recorded with a scanning electron microscope at 30 kV using a scanning‐transmission detector and the image shown in E was recorded with a transmission electron microscope (120 kV) using a side‐mounted CCD camera and 4.1k by 3k pixels. Scale bars in A = 2 µm, B and C = 500 nm, D and E = 200 nm.

This approach is also helpful in case of infections that appear in tissue which is normally not affected. During the last monkeypox (mpox) outbreak in Europe, patients showed up in hospital and complained about intense pain in the rectal region. Coloscopy revealed that larger regions of the upper anal channel were affected by inflammation. However, biopsies from the region, which is histologically similar to the colon, were not significant in histopathology, besides infiltrates of immune cells and a slight de‐arrangement of epithelial cells of some of the colon crypts (invaginations of the surface epithelium). Thin section EM of the corresponding regions inspected by histopathology demonstrated that in in some of the crypts epithelial cells are infected with poxviruses and produce significant amount of new virus particles.^72^ However, the cells did not show large inclusion bodies which are clearly distinct from the rest of the cytoplasm which explains the missing of the typical staining pattern in histopathology. Moreover, although the cells produced many viruses and showed significant signs of cell death (Figure 6A and B), larger necrotic areas were not clearly detectable and therefore the epithelial de‐arrangement was not obvious in histopathology.

Diagnostic EM of the mpox case revealed additional information which is relevant to understand the pathogenesis of the disease. The cellular tropism of orthopoxviruses, such as the monkeypox virus (MPXV), usually is broad which could be proved for MPXV in the patient tissue. Infection and productive replication could be found not only in the enterocytes, but also in endothelial cells, smooth muscle cells and fibroblasts. Most strikingly, the neutrophilic granulocytes, which were present at high number among the infected enterocytes, were also infected but never showed any sign of replication (Figure 6A). The morphology of the virus particles found in the cytoplasm of neutrophilic granulocytes was identical with mature virus particles (Figure 6C) or resembled orthopoxvirus particles that are trapped in the de‐coating step of their infection process (Figure 6D and E) which implies that neutrophilic granulocytes can somehow stop the infection before it becomes productive.

This last example illustrates that extending histopathology by ultrastructural pathology is important in disease cases which are uncommon and it again demonstrates that it is necessary to consider the cellular level and not only the molecular. Thus, combining these two approaches should be standard in studying the pathogenesis of an emerging or under‐studied disease which is the basis for developing treatment and also prevention. Several studies from the literature support this conclusion and few of them are exemplarily mentioned in the following. A combined histo‐ and ultrapathological study on the human immunodeficiency virus (HIV) demonstrated that in the late stage of the disease macrophages serve as a highly productive host cell for the virus in patients which is promoted by opportunistic co‐infections.^110^ In a rare case of a Marburg virus infection, diagnostic EM could clearly demonstrate, by showing virus factories in the respective cells, the broad cellular tropism of the virus in the patient which was unknown at the time of investigation.^111^ More recently, hepatitis E virus particles could be clearly identified in the seminal fluid from chronically HEV‐infected patients which directed attention to a sexual transmission of this worldwide prevalent virus.^81^ And finally, during the COVID‐19 pandemic, which demonstrated the complexity of infectious diseases at all its levels, diagnostic electron microscopy could show that a persistent viral lung infection and replication was not promoting the Acute Respiratory Distress Syndrome (ARDS) in fatal cases although virus RNA and protein were present.^71^

## 4 OUTLOOK

The recognition and investigation of microbial pathogens related to infectious diseases by electron microscopy will be necessary in the next decades because infectious diseases will be present and, most likely, no other method will be available soon to directly detect the infectious unit and to visualise its ‘behaviour’ in the patient. Certainly, most of our knowledge will derive from the amazing molecular technologies developed during the last years, such as single‐cell transcriptomics or spatial proteomics. However, to fully understand the pathogenesis of an infectious disease, it is necessary to put all available information into context. The complex interplay of infection and host response, as seen in the COVID‐19 pandemic, with the many post‐COVID cases, is only one example for the need of a multimethod approach in the research of infectious diseases. Studying infection of explanted patient tissue might help to address some of the open questions experimentally, as has been shown in some examples for lung and skin tissue.^112^, ^113^, ^114^

While routine lab diagnostics of infectious diseases will likely apply more generic approaches (e.g. sequencing techniques) in future, diagnostic EM will still be necessary for those few cases in which a high certainty of the diagnostic result is required (e.g. outbreaks or other emergencies). The extraordinary sensitivity of molecular diagnostics is one of their disadvantages because it is also sensitive for contaminations or the molecular background of the samples which may impair a correct recognition of the pathogen present.

With ongoing developments of synthetic biology,^115^, ^116^ we also have to expect the emergence of pathogens with completely new genome assemblies and features. In such scenarios, it would be definitely necessary to apply completely independent methods in parallel for a reliable identification and study of the new pathogen^117^ because the molecular fingerprint would most likely indicate the presence of various pathogens and likely miss or underestimate new sequences. It is not farfetched that this argumentation is also valid for the detection and the study of emerging infectious diseases caused by naturally evolved pathogens. The fast change of our environment due to the overusage of natural resources and climate change is a strong selection force and might generate new pathogens with unusual features.

Diagnostic EM will also evolve in the next decades and profit from the ongoing improvements in resolution and automation (e.g. machine learning, large‐field and array imaging) of microscopy. Scanning EM will be used for more applications in diagnostic EM although the practical resolution and speed of imaging still must be increased to achieve the practical requirements for all use cases of diagnostic EM related to infectious diseases. The true challenge for the field is to preserve and, perhaps, even to expand the ability to detect and to identify microbial pathogens using their structural signatures because this is essential to train people and machines for the challenges of the future.

## Supporting information

Supporting information

**Video S1** Zoom and pan in a large‐field EM data set recorded from an ultrathin section through the skin of a *Treponema*‐infected skin of a nonhuman primate. The section was scanned with a field‐emission electron microscope and an in‐lens back‐scattered electron detector using 23 by 29 tiles at a pixel size of 11 nm.
