# Supplementary material for: Diagnostic electron microscopy in human infectious diseases – Methods and applications
Source: J Microsc. 2024 Nov 19;299(3):186–205. doi: 10.1111/jmi.13370 (PMC12352021; doi:10.1111/jmi.13370)
Supplement: Supplementary file 1 — Supporting information [file JMI-299-186-s001.pdf]

Diagnostic Electron Microscopy in Human Infectious Diseases – Methods and Applications

- Supporting Information -

Michael Laue

Advanced Light and Electron Microscopy, Centre for Biological Threats and Special Pathogens (ZBS 4), Robert Koch Institute, Germany

Dr. Michael Laue (ORCID: 0000-0002-6474-9139)
Advanced Light and Electron Microscopy
Centre for Biological Threats and Special Pathogens 4 (ZBS 4)
Robert Koch Institute
Seestr. 10
D-13353 Berlin
Germany
lauem@rki.de
+49(0)30 18754 2675

Contents

1. Link list to protocols for diagnostic electron microscopy (EM) related to infectious diseases 3
   1.1 Negative Staining EM 3
   1.2 Thin section EM 3
   1.3 Microscopy 4
   1.4 Reference Images 4
2. Selected and commented references on diagnostic EM related to infectious diseases 4
3. Preparation of patient samples before EM sample preparation 7
4. Thin section EM – Step-by-step protocol of manual epoxy-resin embedding 9
5. Thin section EM – Step-by-step protocol of machine-assisted epoxy-resin embedding (Leica TP) 11
6. Thin section EM – De-paraffinization and preparation of paraffin-embedded samples 13
7. Thin section EM – Agarose embedding of suspensions 14

1. Link list to protocols for diagnostic EM related to infectious diseases (mainly from our lab)

1.1 Negative Staining EM

Negative staining – Protocols and detection limit
Laue (2010) *Meth. Cell Biol.* https://doi.org/10.1016/S0091-679X(10)96001-9

Laue & Bannert (2010) *J. Appl. Microbiol.* https://doi.org/10.1111/j.1365-2672.2010.04737.x

Negative staining – Step-by-step protocol
https://zenodo.org/record/1468676

Negative staining – Step-by-step video

https://zenodo.org/record/1468500

Negative staining – Airfuge particle enrichment – step-by-step protocol

https://doi.org/10.5281/zenodo.12731515

Negative staining – Airfuge particle enrichment – step-by-step video

https://doi.org/10.5281/zenodo.12705300

Negative staining – Particle enrichment by filtration
Beniac et al. (2014) *Viruses* https://doi.org/10.3390/v6093458

Goldin et al. (2016) *Sci. Rep.* https://doi.org/10.1038/srep26516

Negative staining – Inactivation of virus suspensions
Möller et al. (2015) *Viruses* https://doi.org/10.3390/v7020666

Immuno-negative staining – Protocol
Laue (2010) *Meth. Cell Biol.* https://doi.org/10.1016/S0091-679X(10)96001-9

Immuno-negative staining – Aggregation by cross-linking
Lavazza et al. (2015) *Viruses* https://doi.org/10.3390/v7052683

1.2 Thin section EM

Thin section EM – Protocol of standard and rapid embedding
Laue (2010) *Meth. Cell Biol.* https://doi.org/10.1016/S0091-679X(10)96001-9

Thin section EM of (patient) virus suspensions
Laue et al. (2023) *Virol. J.* https://doi.org/10.1186/s12985-023-01981-9

Thin section EM – Step-by-step protocol of manual embedding
see page 9 of this document

Thin section EM – Step-by-step protocol of machine-assisted embedding (Leica TP)
see page 11 of this document

Thin section EM – Step-by-step protocol of de-paraffinization
see page 13 of this document

Thin section EM – Step-by-step protocol of agarose embedding of suspensions
see page 14 of this document

1.3 Microscopy

Diagnostic EM with Low-voltage SEM, STEM or TEM
Möller et al. (2020) *J. Histochem. Cytochem.* https://doi.org/10.1369/0022155420929438

1.4 Reference images for object recognition

VirusExplorer DEM – A reference database for diagnostic EM of viruses
Möller & Laue 2021 https://zenodo.org/record/4897236

VirusExplorer DEM – Image catalogue (PDF version of the images included in the database)
Möller & Laue 2021 https://zenodo.org/record/4900042

2. Selected references on diagnostic EM related to infectious diseases

2.1 Overview // Reviews

Biel & Gelderblom (1999) *J. Clin. Virol*. https://doi.org/10.1016/s1386-6532(99)00027-x

Curry et al. (2006) *Micron* https://doi.org/10.1016/j.micron.2005.10.001

Curry, A. (2013) Microbial ultrastructure. *In: Diagnostic electron microscopy: A practical guide to interpretation and technique. Eds.: J.W. Stirling, A. Curry, B. Eyden. Chichester: Wiley*. p181-219.

Gentile & Gelderblom (2014) Electron microscopy in rapid viral diagnosis: an update. *The New Microbiologica*, *37*(4), 403–422.

Goldsmith (2014) *Viruses* https://doi.org/10.3390/v6124902

Goldsmith et al. (2013) *Emerg. Infect. Dis.* https://doi.org/10.3201/eid1906.130173

Goldsmith & Miller (2009) *Clinical Microbiology Reviews* https://doi.org/10.1128/CMR.00027-09

Hazelton & Gelderblom (2003) *Emerging Infectious Diseases* https://doi.org/10.3201/eid0903.020327

Miller (1986) *The Journal of Microscopy Technique* https://doi.org/10.1002/jemt.1060040305

Richert-Pöggeler et al. (2019) *Front. In Microbiol.* https://doi.org/10.3389/fmicb.2018.03255

Roingeard (2008) *Biology of the Cell* https://doi.org/10.1042/BC20070173

Roingeard et al. (2019) *Reviews in Medical Virology* https://doi.org/10.1002/rmv.2019

2.2 Methods (without references mentioned above)

Electron microscopy sample preparation

Bozzola & Russell (1998) Electron microscopy. Principles and techniques for biologists. *Jones and Bartlett Publishers*. *SEM & TEM basic methods. Perfect for beginners with many images on the tools and procedures, including a chapter on image interpretation.*

Dykstra, M.J., Reuss, L.E. (2003) Biological electron microscopy: theory, techniques, and troubleshooting. *Kluwer Academic/Plenum Publishers*. *Presents sample preparation protocols (thin section EM) for different biological samples.*

Hayat, M.A. (2000) Principles and Techniques of Electron Microscopy: Biological Applications (4th Edition). *Macmillan Press Houndmills, Basingstoke, London*.

Kuo, J (2007) Electron microscopy. Methods and protocols. Second edition. *Humana Press*. *Different chapters on various methods with a lot of suitable information.*

Kuo, J (2014) Electron microscopy. Methods and protocols. Third edition. Humana Press. *Somewhat different content from the preceding edition. Makes sense to have both.*

Maunsbach, A.B., Afzelius, B.A. (1999) Biomedical Electron Microscopy: Illustrated Methods and Interpretations. *Academic Press, San Diego*. *Includes comparisons of the effects of preparation variables. Many excellent images.*

Electron microscopy – Fixation

Biel, S.S.; Gelderblom, H.R. (1999) Electron microscopy of viruses. *In: Virus Cell Culture. Ed.: Alan Cann. Oxford University Press.* p.111-147. *One section of this fine method collection is on fixation.*

Griffiths, G. (1993) Fine structure immunocytochemistry. *Heidelberg, Berlin: Springer*. *Chapter 3.*

Hayat, M.A. (1981) Fixation for electron microscopy. *New York, London, Toronto: Academic Press*. *A comprehensive monograph on the topic with many details.*

Humbel, B.M.; Schwarz, H.; Tranfield; E.M.; Fleck, R.A. (2019) Chemical fixation. *In: Biological Field Emission Scanning Electron Microscopy. Eds.: Roland A. Fleck, Bruno M. Humbel. Wiley*. p.191-221. *A very good summary on the topic.*

Negative Staining

Biel, S.S.; Gelderblom, H.R. (1999) Electron microscopy of viruses. *In: Virus Cell Culture. Ed.: Alan Cann. Oxford University Press.* p.111-147. *Detailed description of negative staining protocol for diagnostic EM, including immuno-negative staining and solid-phase immuno-EM techniques.*

Harris & De Carlo (2014) *In: Electron Microscopy. Methods and Protocols.* https://doi.org/10.1007/978-1-62703-776-1_11 *General protocols for negative staining EM.*

Harris (1997) Negative Staining and Cryoelectron Microscopy. *BIOS Scientific Publishers.*
Detailed monograph with many images/photographs of technical procedures/machines and examples
Hayat & Miller (1990) Negative Staining. *McGraw-Hill Publishing Company.*
Detailed monograph with a separate chapter on virology.

Negative staining – Electron tomography (ET)

Fera et al. (2012) *Microsc. Microanal.* https://doi.org/10.1017/S1431927611012797

Mast & Demeestre (2009) *Diagnostic Pathol.* https://doi.org/10.1186/1746-1596-4-5

ET seems to be a somewhat exotic approach for diagnostic purposes, but could be helpful to reveal detailed information on overlaying structures. Modern microscopes allow to record fast tilt series and the post-processing is facilitated by open source software.

Thin section EM

Biel, S.S.; Gelderblom, H.R. (1999) Electron microscopy of viruses. *In: Virus Cell Culture. Ed.: Alan Cann. Oxford University Press.* p.111-147. *Detailed description of thin section EM for diagnostic EM, including immuno-staining for thin section EM.*
Bretschneider et al. (1981) *American Journal of Clinical Pathology* https://doi.org/10.1093/ajcp/76.4.450
Protocol for embedding of paraffin-sections on the slide (capsule embedding).
Doane et al. (1974) *Appl. Microbiol*. https://doi.org/10.1128/am.27.2.407-410.19
A simple two-hour embedding protocol for thin section EM.
Estrada et al. (2005) *Microscopy Today*. https://doi.org/10.1017/S1551929500053773
Protocol for embedding of paraffin-sections on the slide (thin layer embedding).
Graham & Orenstein (2007) Nature Prot. https://doi.org/10.1038/nprot.2007.304
Protocols for general ultrastructural pathology, including de-paraffinization.
van den Bergh Weerman, M. A., & Dingemans, K. P. (1984). *Ultrastructural Pathology* https://doi.org/10.3109/01913128409141854
Protocol for de-paraffinization of tissue extracted from a paraffin block.

Thin section EM – Large-scale electron microscopy

Dittmayer et al. (2021) *Microscopy and Microanalysis* https://doi.org/10.1017/S1431927621011958
Detailed description of ultramicrotomy, STEM large-field imaging and data post-processing of thin sections for ultrastructural pathology.
Kataoka et al. (2019) *J. Virol.* https://doi.org/10.1128/JVI.00644-19
Application of large-scale SEM of thin sections on a case of lung infection.
Kolotuev (2024) *Journal of Microscopy* https://doi.org/10.1111/jmi.13217
Review on SEM imaging of thin (serial) sections on solid supports and their suitability for cell and tissue research.
Kuipers et al. (2015) *Experimental Cell Research* https://doi.org/10.1016/j.yexcr.2015.07.012
One of the first papers on large-scale SEM of thin sections and its perspectives. See also http://www.nanotomy.org/
Müller et al. (2022) *Lancet* https://doi.org/10.1016/S0140-6736(22)01969-9
Application of large-scale STEM of thin sections on a case of Monkeypox virus infection.

Thin section EM of autopsy samples – Recommendations / Limitations

Bachofen & Weibel (1977) *Am. Rev. Resp. Dis.* https://doi.org/10.1164/arrd.1977.116.4.589
Good preservation of lung tissue after in situ fixation of tissue.
Cortese et al. (2022) *Virchows Archiv* https://doi.org/10.1007/s00428-022-03308-5
Sufficient preservation of lung tissue from deceased patients by taking cryo-biopsies
Krasemann et al. (2023) *EBioMedicine* https://doi.org/10.1016/j.ebiom.2022.104193
A review on the histo- and ultrapathology of COVID-19 autopsy samples.

Immuno-EM

Griffiths (1993). Fine-Structure Immunocytochemistry. Springer. *Covers all aspects of on-section immuno-EM, including chapters on fixation and evaluation of labelling experiments.*
Hayat & Eaton (1993). Immuno-gold Electron Microscopy. CRC Press. *Broad scope of methods, including the immune-aggregation methods for negative staining EM and immunolabeling of replicas.*
Palmer, E.L.; Martin, M.L. (1993) Immune complexing. *In: Immuno-gold electron microscopy in virus diagnosis and research. Eds.: A.D. Hyatt; B.T. Eaton. Boca Raton, Ann Arbor, London, Tokyo: CRC Press. p.3- 24.*
Overview about immuno-aggregation methods.

2.3 Reference books for diagnosing pathogens

Dickerson, R.G. (2000) Diagnostic Electron Microscopy: A Text / Atlas (2nd Edition). Springer-Verlag, J Gaku-Shoin, New York/Tokyo. *Selected images from viral, bacterial, fungal and parasitic species in tissue (thin section EM).*

Doane, F.W.; Anderson, N. (1987) Electron Microscopy in Diagnostic Virology. A Practical Guide and Atlas. Cambridge University Press. *Description of methods. Reference images from negative staining and thin sections.*

Madeley, C.R., Field, A.M. (1988) Virus Morphology (2nd Edition). Churchill Livingston Edinburgh (ISBN 0-443 02 7846). *The reference for negatively stained viruses.*

Palmer, E. L. and Martin, M. L. (1988) Electron Microscopy in Viral Diagnosis. CRC Press, Boca Raton. *Reference images from negative staining and thin sections.*

Palmer, E. L., Martin, M. L. (1985) An Atlas of Mammalian Viruses. CRC Press, Boca Raton. *Images of negatively stained viruses.*

Stirling, J.W.; Curry, A.; Eyden, B. (Eds.) (2013) Diagnostic electron microscopy: A practical guide to interpretation and technique. Wiley, Chichester. *In chapter 6 and 16 images of relevant viruses, bacteria, fungal and parasitic species are shown.*

2.4 Reference books for the ultrastructure and pathology of tissue and cells

Connor, Chandler, Schwartz, Manz, Lack (1997) Pathology of Infectious Diseases. Appelton & Lange. Two Volumes. *Mainly histology and presentation of symptoms but also several EM images (virology chapter, microsporidia chapter).*

Hossler (2014) Ultrastructure Atlas of Human Tissues. Wiley Blackwell.
Atlas of the cellular ultrastructure of human tissue. Overview (including SEM images) and details

Pavelka & Roth (2010) Functional Ultrastructure. Springer.
Atlas of the cellular ultrastructure of human/mammalian tissue with a focus on functional aspects.

Procop & Pritt (2015) Pathology of Infectious Diseases. Elsevier Saunders. *Mainly presentation of cases (symptoms/histopathology) with few EM images. Interesting collection*

Rhodin (1974) Histology. A Text and Atlas. Oxford University Press.
Atlas of the cellular ultrastructure of human/mammalian tissue. More details than overviews.

Rhodin (1975) An Atlas of Histology. Oxford University Press.
Detailed, large format, atlas of the ultrastructure of human/mammalian tissue from overviews to details. The best atlas I am aware of.

3. Preparation of patient samples before EM sample preparation

Blood (collected in EDTA or Heparin-coated vials)

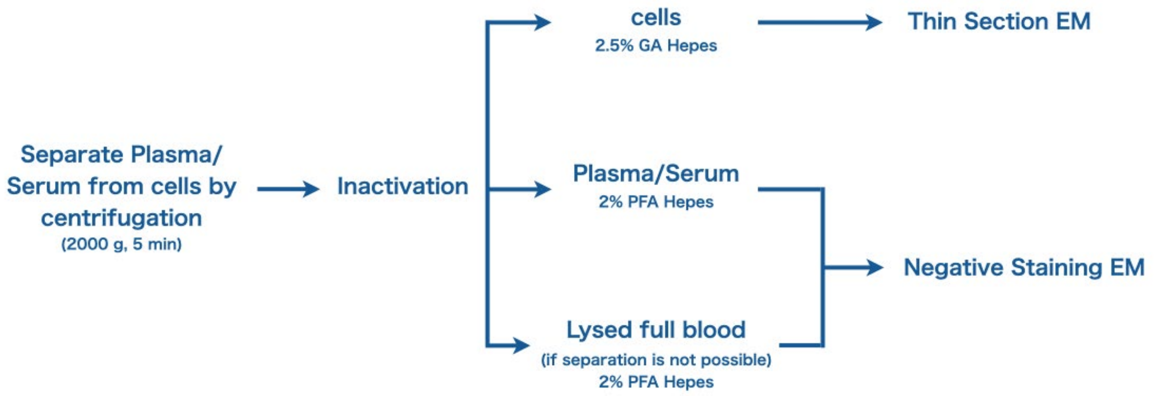

Stool

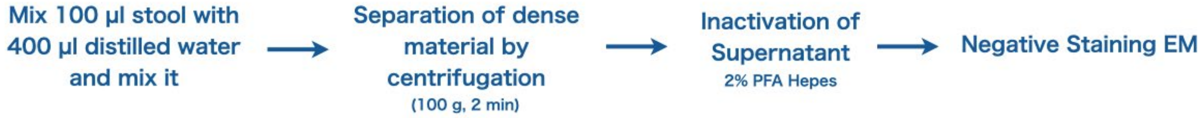

Urine

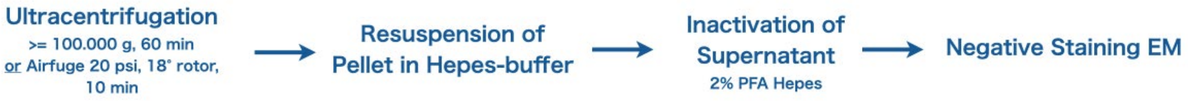

Swabs (eSwabs from Copan – discard Amies transport medium before use)

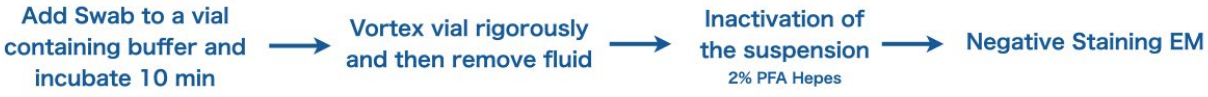

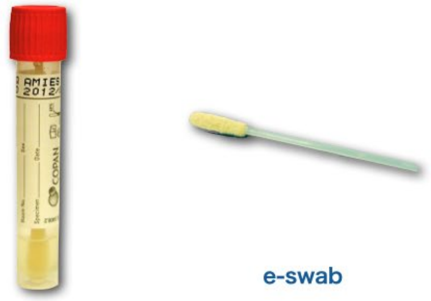

Crust / tissue material (unfixed)

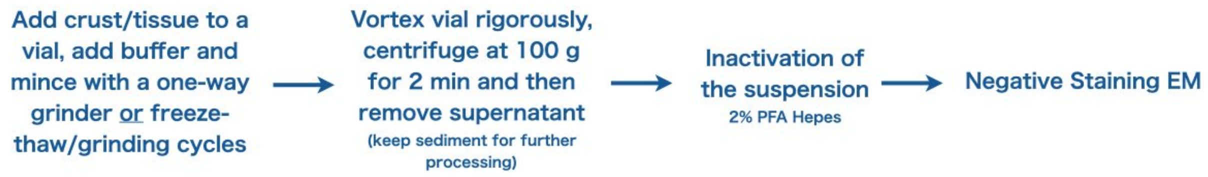

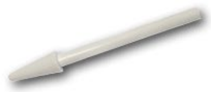

grinder which fits in reaction vial

Sputum

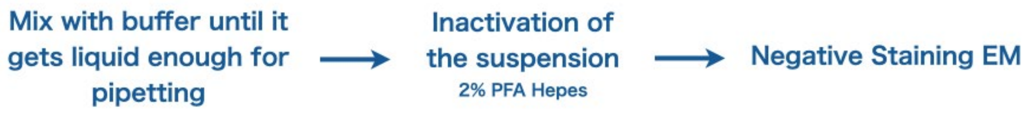

All inactivation steps are optional. See section 2.4 of the main text.

4. Thin section EM – Step-by-step protocol of manual resin embedding

-------------- **Materials** --

(1) Chemically fixed samples (1-2 mm sample size in all directions)

(2) HEPES buffer, 0.05 M, pH 7.2

(3) Osmium tetroxide, 4% [w/v] in double-distilled water (stock solution)

(4) Tannic acid (Mallinckrodt, No. 1746)

(5) Na_2SO_4, 1% [w/v], in HEPES buffer

(6) Uranyl acetate, 2% [w/v], in double-distilled water

(7) Ethanol, 100%

(8) Acetone

(9) Glycidyl ether 100 (Serva, No. 21045.02)

(10) Methylnadic anhydride (MNA; Serva, No. 29452.03)

(11) Dodenyl succinic anhydride (DDSA; Serva, No. 20755.02)

(12) 2,4,6-Tris[dimethylaminomethyl]phenol (DMP; Serva, No. 36975.01)

(13) Oven for polymerization

(14) Desktop centrifuge

(15) Glas vials with tight fitting plastic lid (Agar Scientific G284)

(16) Mould for embedding (e.g. Pelco 106)

(17) Plastic Pasteur pipettes (Sarstedt, No. 86.1171)

(18) Optional: Rotator or horizontal shaker

------------- **Preparation** --

(19) Dilute osmium tetroxide stock solution to 1% with double-distilled water

(20) Prepare tannic acid solution: 0.1% [w/v] in HEPES buffer

(21) Centrifuge uranyl acetate solution directly before use with a desktop centrifuge (max. speed, 10 min)

(22) Prepare ethanol dilutions for dehydration

(23) Prepare Epon resin: 23.52 g glycidyl ether, 12.35 g DDSA, 14.13 g MNA, 0.65 g DMP. Note: mix the first three thoroughly before adding of the accelerator.

(24) Prepare Mixtures of Epon and Acetone: 1+1, 2+1, 3+1

(25) Cast a thin layer of resin in moulds and polymerize it overnight. Creates bottom for embedding the samples.

-------------- Procedure --

All incubations were at RT if not stated otherwise. The entire preparation is performed in a fume hood. Suitable gloves for personal protection are used. Samples are slightly moved on a shaker or rotator.

(26) HEPES buffer, 3x, 10 min each

(27) Osmium tetroxide, 1%, 60 min

(28) Double-distilled water, 2x, 10 min each

(29) HEPES buffer, 1x, 5 min

(30) Tannic acid, 0.1%, in HEPES buffer, 30 min

(31) Na_2SO_4, 1%, in HEPES buffer, 2x, 10 min each

(32) Double-distilled water, 3x, 10 min each

(33) Uranyl acetate, 2%, 120 min

(34) Double-distilled water, 2x, 10 min each

(35) Ethanol, 30%, 10 min

(36) Ethanol, 50%, 10 min

(37) Ethanol, 70%, 10 min

(38) Ethanol, 90%, 10 min

(39) Ethanol, 96%, 10 min

(40) Ethanol, 100%, 2x, 10 min each

(41) Acetone, 2x, 5 min each

(42) Acetone/Epon 1+1, 60 min

(43) Acetone/Epon 1+2, 60 min

(44) Acetone/Epon 1+3, overnight with removed lid in a fume hood

(45) Transfer samples in fresh epoxy resin and incubate for a few hours

(46) Embed the samples in fresh resin filled in a suitable mould with or without prepared resin layer on the bottom

(47) Polymerisation at 60 °C for at least 1 day

5. Thin section EM – Step-by-step protocol of machine-assisted resin embedding

Step	Solution/Mixture/Solvent	Duration of incubation [min]	Temperature	Tissue processor
1	HEPES buffer, 0.05 M	5	RT	no
2	HEPES buffer, 0.05 M	5	RT	no
3	HEPES buffer, 0.05 M	5	RT	no
4	Osmium tetroxide, 1% in water	60	RT	no
5	Distilled water	5	RT	no
6	Distilled water	5	RT	no
7	Distilled water	5	RT	no
8	Tannic acid, 0.1% in 0.05 M HEPES buffer	30	RT	yes
9	Na_2SO_4 1% in 0.05 M HEPES buffer	10	RT	yes
10	Na_2SO_4 1% in 0.05 M HEPES buffer	10	RT	yes
11	Distilled water	10	RT	yes
12	Distilled water	10	RT	yes
13	Distilled water	10	RT	yes
14	Uranyl acetate, 2% in distilled water	120	RT	yes
15	Ethanol, 30%	30	RT	yes
16	Ethanol, 50%	30	RT	yes
17	Ethanol, 70%	30	RT	yes
18	Ethanol, 95%	60	RT	yes
19	Ethanol, abs.	60	RT	yes
20	Ethanol, abs.	60	RT	yes
21	Acetone	30	RT	yes

22	Acetone	30	RT	yes
23	Acetone / Epon 2+1	180	RT	yes
24	Acetone / Epon 1+1	180	RT	yes
25	Acetone / Epon 1+3	180	RT	yes
26	Epon	120	RT	yes
27	Epon	180	RT	yes
28	Epon	240	RT	no
29	Epon - final embedding in silicone moulds	-	RT	no
30	Polymerization	2 days	60 °C	no

Tissue processor = Leica EM TP (Leica Microsystems)

RT = room temperature

All chemicals as indicated in the manual protocol.

6. Thin section EM – De-paraffinization and preparation of paraffin-embedded samples

-------------- **Material** --

(1) Skin tissue punch (3 to 5 mm diameter)

(2) Skalpel

(3) Xylene

(4) HEPES, 0.05M, pH 7.2

(5) Fixative (e.g. 1% PFA 2.5% glutaraldehyde in HEPES buffer)

(6) Ethanol

(7) Horizontal shaker or rotator

(8) Glas vials with tight plastic lid (e.g Agar Scientific G284)

------------- **De-Paraffinisation** ---

(9) Extract region of interest from the paraffin block by using a tissue punch and/or scalpel. Reduce block further, if possible. Remove all Paraffin which does not contain material.

(10) Xylene, 3x, 10 min each, on a shaker or rotator

(11) Ethanol (100%), 2x, 5 min each

(12) Ethanol (96%), 5 min

(13) Ethanol (70%), 5 min

(14) Ethanol (50%), 5 min

(15) HEPES, 0.05 M, 3x, 1 min each

(16) Immersion in fixative, for at least 1 h, at room temperature

(17) Long time storage at 4-8 °C or direct processing for embedding in resin (see protocols above)

------------- **Remarks** --

To avoid longer processing times in xelene or ethanol, the extracted pieces of tissue should be as small as possible. If a larger area needs to be sampled, the tissue piece should be thin (<= 1 mm). As a consequence, incubation time of the several steps of the manual dehydration and infiltration with resin can be reduced: uranyl acetate (0.5 h), ethanol dehydration (5 min each step), acetone/epon infiltration (0.5 h each step, except 1+3 which is overnight).

7. Thin section EM – Agarose embedding of suspensions in thin gel chambers

-------------- **Material** --

(1) Slides (76 x 26 mm)

(2) Spacer (Gene Frame, 125 µl; ABgene AB-0578 <u>or</u> coverslips <u>or</u> thin transparent tape, e.g. Tesafilm)

(3) Document clamps (see Figures 2, 3)

(4) Ethanol for cleaning

(5) Water, de-ionized

(6) Low-melting point Agarose (3% in de-ionized water; Sigma A9414)

(7) Alcian blue, 1% in de-ionized water (optional)

(8) Glutaraldehyde, 1-2.5 % in buffer (optional)

(9) Reaction vials, 0.5 ml and 2 ml

(10) Heated water bath with rack for reaction vials

(11) Ice

(12) Vortex mixer

(13) Desktop centrifuge

(14) Microliter pipette (100-200 µl) with tips

(15) Razor blade

(16) Bunsen burner or microwave for boiling the agarose solution

------------- **Preparation** ---

Slides

(17) Cleaning the slides with ethanol.

(18) Adhere gene frame to one slide by removing the frame from the large area protective sheet and pressing it firmly on the slide. This procedure generates a low chamber on the slide. Leave the protective sheet on the upper side of the gene frame (Fig. 1). **Alternative:** Prepare a thin gel chamber by using a coverslip or layers of transparent tape as a spacer between two slides clamped by the document clamps (Fig. 3).

Water bath

(19) Heat water bath to 40°C.

(20) Boil the agarose solution briefly by using a flame or microwave. Use a Vortex mixer to mix solution after cooling down below boiling point.

(21) Cut pipette tips with razor blade. Insert them in 2 ml reaction vials and place them in the heated water bath for warming.

(22) Warm the sample suspension or pellet in the water bath.

Sample

Optionally, but usually helpful to increase concentration: centrifugation with a desktop centrifuge or ultracentrifuge.

------------- **Procedure** ---

(23) Estimate volume of sample suspension or pellet.

(24) Mix agarose solution with the Vortex mixer and add it to the sample pellet or solution (1:1 [v/v]) by using a pre-warmed pipette tip. Resuspend and mix the agarose/sample mixture with the pipette and the Vortex mixer.

(25) Warm the prepared slides (e.g. at the rim of the water bath). Fill the agarose/sample mixture into the gene frame chamber on the pre-warmed slide using a pre-warmed pipette tip. Place the second slide on top of the gene frame and clamp it with two document clamps (Fig. 2). **Al-ternative:** Fill the agarose/sample mixture in the gel chamber which was assembled from two slides with alternative spacers. Add the tip to the thin slit-like opening of the chamber and slowly release the suspension from the tip. Capillary forces will suck the fluid between the two slides (Fig. 3).

(26) Cool the slide on ice for 5 min.

(27) Open the gel chamber by removing the clamps and by carefully shifting the upper slide from the gene frame.

(28) Cut the thin gel into small pieces suitable for embedding.

(29) Optional: Stain the gel pieces with Alcian blue (1:10 diluted in water) for a couple of seconds. Wash the gels in water.

(30) Store the gels in fixative (glutaraldehyde) or buffer or use them directly for embedding.

------------- **Remarks** ---

The smallest sample volume which can be prepared reliably is 10 µl. The use of positive displacement pipettes facilitate the pipetting of small volumes of the agarose and of agarose/sample mixtures.

------------- **Figures** ---

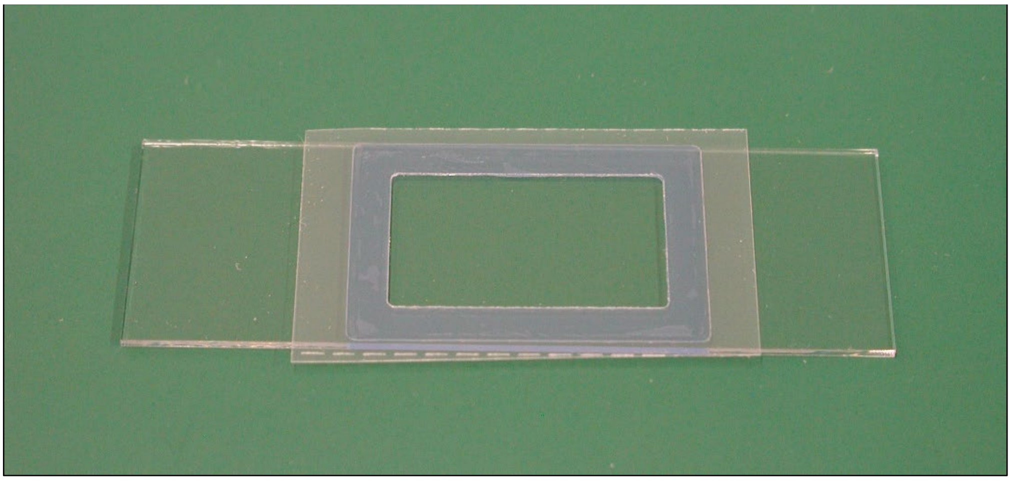

Fig. 1: Open chamber formed by the Gene Frame spacer on a slide. The upper cover remains on the Gene frame to allow opening of the chamber for removing the gel.

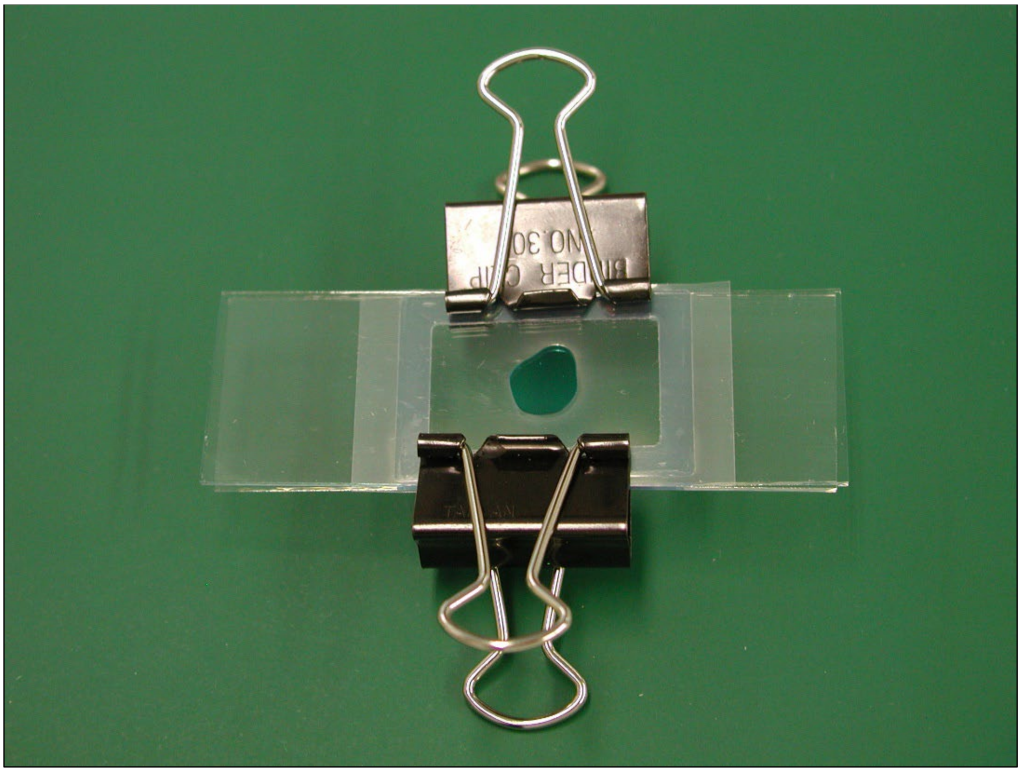

Fig. 2 Closed slide chamber with a small area gel in the middle.

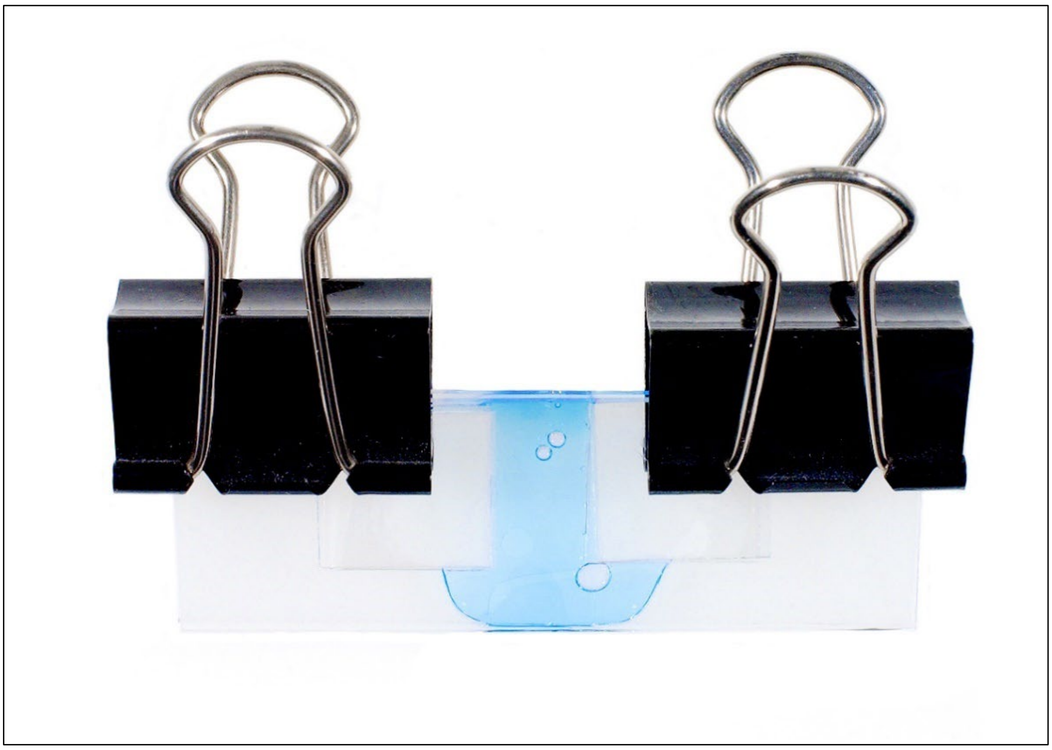

Fig. 3 Alternative assembly of the gel chamber by using coverslips as spacer. The gel was stained by Alcian blue.
